# Lactylation modification of HIF-1α enhances its stability by blocking VHL recognition

**DOI:** 10.1186/s12964-025-02366-x

**Published:** 2025-08-04

**Authors:** Chengyu Li, Chen Fu, Wenhan Zhou, Hongmin Li, Zhaojun Liu, Gang Wu, Tong He, Ming Shen, Honglin Liu

**Affiliations:** College of Animal Science and Technology, Nanjing Agricultural University, Weigang 1, Nanjing, 210095 China

**Keywords:** HIF-1α, Lactate, Lactylation, Ubiquitination, Hydroxylation, VHL, Degradation

## Abstract

Hypoxia-inducible factor 1α (HIF-1α) is a master regulator of cellular adaptation to hypoxia. Although prolyl hydroxylation-mediated degradation via the von Hippel–Lindau (VHL) ubiquitination complex is a well-established regulatory mechanism, the role of lactate-induced posttranslational modifications in HIF-1α stabilization remains incompletely understood. Here, we demonstrate that lactate induces lysine lactylation of HIF-1α at distinct residues across species—specifically, K644 in mice and K12 in humans and pigs—to increase protein stability by impairing VHL recognition. Mass spectrometry and mutagenesis analyses revealed that lactylation at these sites reduces K48-linked ubiquitination and proteasomal degradation, even when HIF-1α is hydroxylated. Structural modeling and functional assays revealed that lactylation sterically hinders VHL binding without affecting hydroxylation. Notably, lactylated HIF-1α exhibited increased transcriptional activity, as evidenced by increased promoter occupancy and upregulation of hypoxia-responsive genes (Vegfa, Glut1). Cross-species comparisons highlighted evolutionary divergence in lactylation sites while preserving the functional conservation of this modification. Our findings reveal that lactylation is a universal regulatory mechanism that overrides classical hydroxylation-dependent degradation, expanding our understanding of metabolic control over hypoxic signaling.

**Supplementary Information:**

The online version contains supplementary material available at 10.1186/s12964-025-02366-x.

## Introduction

One critical factor in the physiological response to hypoxia is hypoxia-inducible factor-1 (HIF-1). HIF-1 is a heterodimeric protein composed of two subunits: HIF-1α and HIF-1β (also known as ARNT) [1]. Under normoxic conditions, the HIF-1α protein is continually synthesized, but it undergoes rapid degradation in the presence of oxygen. The main mechanism involves the posttranslational hydroxylation of HIF-1α at proline residues 402 and 564 via oxygen-dependent prolyl hydroxylases (PHDs). The hydroxylated proline residues of HIF-1α are then recognized by the E3 ubiquitin ligase adaptor von Hippel–Lindau (VHL) protein, leading to HIF-1α polyubiquitination and its subsequent degradation by the 26 S proteasome [2, 3]. Under hypoxic conditions, where oxygen is unavailable for the hydroxylation reaction, HIF-1α accumulates and forms a dimer with the oxygen-independent ARNT [4]. The expression of various genes is initiated by stabilizing the HIF-1 complex, which promotes hypoxia adaptation by increasing glucose uptake and activating oxygen-independent ATP generation, angiogenesis, and other processes [5].

As a byproduct of glycolysis, lactate increases when cells require oxygen and ATP levels that exceed their supply, such as during exercise or infection [6]. Although lactate was initially regarded as a byproduct, it is now recognized as an intracellular energy source [7]. Extracellularly secreted lactate also functions as a metabolic intermediate or signaling molecule, regulating processes such as tumorigenesis. A recent study revealed that, within tumors, lactate can upregulate the expression of VEGF and ARG1 in tumor-associated macrophages (TAMs), polarizing them toward the immunosuppressive M2 phenotype that supports tumorigenesis [8]. Notably, the ability of lactate to mediate this process likely relies on the stabilization of HIF-1α [9]. Specifically, when cultured under normoxic conditions, HIF-1α expression in TAMs is stabilized by tumor-derived lactate, leading to the transcription of the *VEGFA* gene. These findings indicate that lactate derived from the tumor microenvironment might facilitate HIF-1α-regulated angiogenesis in a hypoxia-independent manner [9]. In addition to its role in tumor tissues, lactate can trigger osteoblast differentiation and induce fibroblast metabolic reprogramming by stabilizing HIF-1α [10, 11]. However, the precise mechanisms underlying how lactate promotes HIF-1α stability have not been fully elucidated.

Recent research has shown that lactate can exert its biological effects via protein lactylation, which involves posttranslational modification of specific lysine residues in proteins [12]. Zhang et al. demonstrated that lactylation modifications directly respond to lactate levels, acting as crucial sensors for metabolic changes and translating them into enduring gene expression patterns [8]. Other investigations have revealed the associations between lactylation modifications and diverse biological processes, including signal transduction, metabolism, inflammatory responses, and tumorigenesis [12]. Ongoing exploration has led to the discovery of delactylases and lactyltransferases, with SIRT1-3 and HDAC1-3 identified as histone lysine delactylases and P300/MOF/GCN5 identified as lactyltransferases [8, 13–15]. Although lactate enhances HIF-1α stability, our understanding of whether lactylation contributes to this process is limited.

In this study, we identified lysine lactylation as a conserved mechanism for HIF-1α stabilization across mammals, albeit through distinct residues: K644 in mice and K12 in humans and pigs. By combining mass spectrometry, structural modeling, and cross-species mutagenesis, we demonstrate that lactylation at these sites disrupts VHL recognition independently of hydroxylation. This modification not only stabilizes HIF-1α but also amplifies its transcriptional output. Our findings redefine the role of lactate in hypoxic adaptation and establish lactylation as an evolutionarily flexible yet functionally conserved regulatory layer in HIF-1α biology.

## Materials and methods

### Reagents and antibodies

Lactate (Cat. No. L6402), sodium lactate (Cat. No. L7022), and CoCl_2_ (Cat. No. 769495) were purchased from Sigma‒Aldrich. 4-Chloro-α-cyanocinnamic acid (CHC; Cat. No. S8612), AZD3965 (S7339), MG132 (Cat. No. S2619), cycloheximide (CHX; Cat. No. S7418), 3-methyladenine (3-MA; Cat. No. S2767), C646 (Cat. No. S7152), DMOG (Cat. No. S7483), and VH298 (Cat. No. S8449) were purchased from Selleck Chemicals. Antibodies against HIF-1α (Cat. No. 36169), HIF-β (Cat. No. 5537), VHL (Cat. No. 68547), TUBA1A (Cat. No. 2125), MYC-tag (2276), His-tag (12698), and mouse IgG (5873) were obtained from Cell Signaling Technology. The antibody against hydroxy-HIF-1α (Cat. No. CVL-PAB0395-0) was obtained from ENZO Life Science. Antibodies against ubiquitin (ab134953), anti-Ubiquitin (linkage-specific K48) (ab140601), rabbit IgG (ab313801), HRP-conjugated goat anti-mouse IgG H&L (ab6789), and HRP-conjugated goat anti-rabbit IgG H&L (ab6721) (ab6789) were obtained from Abcam. Antibodies against LDHA (Cat. No. 66287-1-Ig), LDHB (Cat. No. 66425-1-Ig), and Pan Kac (Cat. No. 66289-1-Ig) were purchased from Proteintech. Antibodies against Pan Kla (Cat. No. PTM-1401RM) were obtained from PTM Bio.

### Sample collection, cell culture, and treatments

Primary-cultured porcine GCs were isolated from the ovaries of mature Duroc–Yorkshire–Landrace sows at a local slaughterhouse by puncturing ovarian follicles with a 10-mL syringe and then cultured in DMEM/F12 (Cat. No. 11320033; Life Technologies) supplemented with 10% fetal bovine serum (FBS; Cat. No. F8687; Sigma‒Aldrich) and 100 U/mL penicillin/streptomycin (Cat. No. 15140122; Gibco) at 37 °C in a humidified atmosphere with 5% CO_2_. NIH/3T3 cells were purchased from the National Collection of Authenticated Cell Cultures (Cat. No. SCSP-515; Cell Bank of Typical Culture Preservation Committee, Chinese Academy of Sciences). These cells originated from a highly contact-suppressed continuous cell line established from NIH Swiss mouse embryo cultures. NIH/3T3, HepG2 and Huh-7 cells were cultured in DMEM (Gibco) supplemented with 10% fetal bovine serum and 100 U/mL penicillin/streptomycin at 37 °C in a humid atmosphere with 5% CO_2_. KGN cells were cultured in DMEM/F12 supplemented with 10% FBS and 100 U/mL penicillin/streptomycin at 37 °C in a humidified atmosphere with 5% CO_2_. For hypoxic exposure, the cells were placed in a modulator incubator in an atmosphere of 94% N_2_, 5% CO_2_, and 1% O_2_. Normoxic conditions were defined as 21% O_2_. For drug administration, the cells were treated with 50 µM cycloheximide (a protein synthesis inhibitor), 5 mM 3-MA (a widely used autophagy inhibitor that suppresses autophagosome formation by inhibiting class III phosphatidylinositol 3-kinase, particularly Vps34), 10 µM MG132 (a proteasome inhibitor), 3 mM CHC (a monocarboxylate transporter inhibitor [MCT]), or 10 µM C646 (an inhibitor for p300) for 2 h before hypoxia exposure. For RNA interference, the cells were transfected with *LDHA* siRNA, *LDHB* siRNA, or scrambled control siRNA for 12 h and then cultured under normoxic or hypoxic conditions for 6 h. For some experiments, the cells were incubated with 100 µM CoCl_2_ or transfected with the HIF-1α (P402A/577A) vector or an empty control plasmid for 12 h under normoxic conditions and then cultured with sodium lactate for 6 h before they were used for the following assay.

### Preparation of nuclear protein lysates

The nuclear extracts were prepared using NE-PER nuclear and cytoplasmic extraction reagents (Thermo Fisher Scientific, 78833) following the manufacturer’s protocol. Briefly, the cells were washed twice with ice-cold PBS buffer and centrifuged at 500 × *g* for 3 min. After adding Cytoplasmic Extraction Reagent I, the lysate was incubated on ice for 10 min, and Cytoplasmic Extraction Reagent II was added to the suspension, followed by centrifugation at 16,000 × *g* at 4 °C for 5 min. The supernatant was collected as the cytoplasmic extract. The insoluble (pellet) fraction was resuspended in nuclear extraction reagent, incubated on ice for 40 min, and then centrifuged at 16,000 × *g* at 4 °C for 10 min. The resulting supernatant was collected as the nuclear extract and utilized for subsequent western blotting or immunoprecipitation (IP) experiments.

### RNA interference

siRNAs specific for *LDHA* and *LDHB* and scrambled control siRNAs (see Supplementary Table S1 for siRNA sequences) were obtained from GenePharma. siRNA transfection was performed using Lipofectamine 3000 (Invitrogen) according to the manufacturer’s instructions.

### Plasmids

The pcDNA3.1 vector subcloned with cDNA of the MYC-tagged HIF-1α wild type (WT) (HIF-1α-WT-MYC), the MYC-tagged HIF-1α K644R mutant (HIF-1α-K644R-MYC) and the 6× His-tagged HIF-1α wild type (His-HIF-1α) was constructed and purchased from KeyGEN. The pcDNA3 vector subcloned with the MYC-tagged HIF-1α P402A/P577A mutant was obtained from Addgene (Cat. No. 44028).

### Immunoprecipitation (IP) and western blotting

For IP, cells washed with PBS (Gibco) were lysed on ice with IP lysis buffer (Cat. No. 26149, Pierce) containing a protease inhibitor cocktail (Cat. No. 04693132001, Roche). Whole-cell lysates (WCLs) were then immunoprecipitated with anti-HIF-1α, anti-MYC, or anti-pan-Kla antibodies. For each IP reaction, we added 5 µL of antibody to 500 µL of cell lysate to achieve a final concentration of 20 ng/µL, and the mixture was incubated at 4 °C overnight. After the addition of 25 µL of protein A/G magnetic beads (Cat. No. 88802, Thermo Fisher Scientific), the mixture was incubated for 1 h at 4 °C. The beads were pelleted with magnetic force, and the supernatant was discarded. The immunoprecipitates were washed with 1× cell lysis buffer, magnetized again to remove the supernatant, eluted with SDS loading buffer (Cat. No. BL502A, Biosharp), and then processed for immunoblotting with the indicated antibodies. For western blotting, the cells were lysed with ice-cold RIPA Lysis Buffer (Cat. No. P0013B, Beyotime) containing a complete protease inhibitor cocktail (Cat. No. 04693132001, Roche), and the protein content was determined using a BCA Protein Assay Kit (Cat. No. P0012, Beyotime). The protein extracts were denatured with SDS in boiling water for 15 min, separated via electrophoresis on a 4–20% Sure PAGE gel (Cat. No. M01012C Genscript), and transferred to PVDF membranes (Millipore) by electroblotting. Nonspecific binding sites were blocked with 5% bovine serum albumin in TBST (Cat. No. T10181, Solarbio) for 1 h. The membranes were then incubated with primary antibodies (1:1000) in blocking solution overnight at 4 °C, followed by incubation with a secondary antibody conjugated with horseradish peroxidase (HRP) (1:2000) for 1 h at room temperature. The bands were visualized using a WesternBright ECL HRP substrate kit (Cat. No. K-12043‐D10, Advansta) following the manufacturer’s directions. The relative expression levels of target proteins were normalized to that of TUBA1A as the loading control, and the signals were quantified using ImageJ software.

### Determination of lactate levels

The lactate levels were determined using a lactate assay kit (A019-2-1, Nanjing Jiancheng Bioengineering Institute). The working and color development solutions were prepared following the manufacturer’s instructions. These solutions were then incubated with the sample for 10 min at 37 °C, followed by the addition of the termination solution. The concentration of chromogenic products was quantified by measuring the absorbance at 570 nm using a TECAN microplate reader. The obtained results were then normalized to the protein concentration of each sample.

### Immunofluorescence

Cells grown on coverslips were subjected to the desired treatments and fixed with 4% paraformaldehyde (Cat. No. P-6148, Sigma‒Aldrich) for 1 h. The cells were washed in PBS (1 mM KH_2_PO_4_, 155 mM NaCl, 3 mM Na_2_HPO_4_–7H_2_O, Gibco) and then permeabilized with 0.5% Triton X-100 (Cat. No. T8787, Sigma‒Aldrich) for 10 min at 4 °C. After blocking with 1% BSA (Cat. No. A3059, Sigma‒Aldrich) for 1 h at room temperature, the cell climbing sheets were stained with an antibody against HIF-1α (1:50) for 1 h at 37 °C. Immunoreactivity was detected by incubation with a goat anti-rabbit IgG (1:500) for 1 h at 37 °C. The nuclei were counterstained with DAPI (Cat. No. D8417, Sigma‒Aldrich) for 20 min. The slides were then visualized under a Zeiss LSM 710 META confocal microscope (Carl Zeiss).

### Quantitative real-time polymerase chain reaction (qRT–PCR)

Total RNA was isolated using TRIzol reagent (Cat. No. 15596026, Invitrogen) and reverse transcribed into cDNA using PrimeScript™ RT Master Mix (Cat. No. 15596026, Takara) according to the manufacturer’s protocol. qRT–PCR was carried out using AceQ qPCR SYBR Green Master Mix (Cat. No. Q111-02, Vazyme) and gene-specific primers (primer sequences are shown in Supplementary Table S2) on an ABI QuantStudio5 system (Applied Biosystems). Melting curves were analyzed to verify amplification specificity. The data were normalized to the expression of the housekeeping gene *TUBA1A*.

### Protein purification and transfection into cells

The His-HIF-1α vector was transfected into cells, and His-tagged HIF-1α protein was subsequently purified using Ni-charged MagBeads (Cat. No. L00295, GenScript) according to the manufacturer’s protocol. Briefly, the cells were lysed in ice-cold lysis equilibration (LE) buffer (50 mM NaH_2_PO_4_, 300 mM NaCl, pH 7.4). The lysate was sonicated on ice using 180 × 1-second bursts with 3-second cooling intervals, followed by centrifugation at 12,000 × g for 15 min at 4 °C to remove cellular debris. The supernatant was collected and incubated with 100 µL of resuspended Ni-charged MagBeads at room temperature with gentle mixing for 30–60 min. The beads were washed three times with wash buffer (50 mM NaH_2_PO_4_, 300 mM NaCl, and 10 mM imidazole, pH 7.4) and eluted three times with elution buffer (50 mM NaH_2_PO_4_, 300 mM NaCl, and 250 mM imidazole, pH 7.4). The purified protein was collected and stored for subsequent experiments. For protein transfection, purified HIF-1α was introduced into cells using the Neon™ Transfection System (Cat. No. MPK5000, Invitrogen). When the cells reached 70–90% confluency, 3 µg of protein was added to each well for transfection. The procedure was as follows: 3 mL of electrolytic buffer was added to the Neon™ tube, which was then inserted into the pipette station. The purified protein was mixed with the cells, and the mixture was aspirated into a Neon™ tip, ensuring that there were no air bubbles. The Neon™ tip containing the sample was inserted into a Neon™ tube, and electroporation was performed according to the device’s programmed settings. Following electroporation, the samples were immediately transferred into prewarmed culture plates containing medium with serum and supplements but without antibiotics. The plates were gently rocked to ensure the even distribution of the cells and then incubated at 37 °C in a humidified CO_2_ incubator for further culture.

### Coomassie brilliant blue staining

The protein extracts were denatured by SDS (Biosharp, BL502A) in boiling water for 15 min and then fractioned by electrophoresis on a 4–20% Sure PAGE gel (GenScript, Nanjing, China). The gel was washed with 100 mL of boiled deionized water for 5 min on a shaking bed for a total of 2–3 washes. After the washing mixture was carefully aspirated, approximately 20 mL of Coomassie Brilliant Blue Rapid Staining Solution (Beyotime, P0017) was added, and staining was performed for 1 h. The Sure PAGE gel was then washed with deionized water for 60 min, and the results were recorded using a camera.

### Ultrahigh-performance liquid chromatography–mass spectrometry analysis

After Coomassie brilliant blue staining, the corresponding gel bands were excised with a razor blade, cut into approximately 1 mm^3^ pieces, and transferred into Eppendorf (EP) tubes filled with deionized water. The samples were lysed with a 4-fold volume of urea lysis buffer, followed by sonication and centrifugation. The resulting supernatant was used for protein concentration determination and subsequent digestion with trypsin. After digestion, the peptides were subjected to acetone precipitation, followed by reduction with DTT and alkylation with IAA. The tryptic peptides were subsequently dissolved in solvent A and directly loaded onto a homemade reversed-phase analytical column (25 cm in length, 100 μm i.d.). The mobile phase consisted of solvent A (0.1% formic acid, 2% acetonitrile/in water) and solvent B (0.1% formic acid, 90% acetonitrile/in water). Peptides were separated with the following gradient: 0–68 min, 6–23% B; 68–82 min, 23–32% B; 82–86 min, 32–80% B; and 86–90 min, 80% B, all at a constant flow rate of 500 nL/min on a vanquish neo-UPLC system (Thermo Fisher Scientific). The separated peptides were analyzed using an Orbitrap Exploris 480 instrument with a nanoelectrospray ion source. The applied electrospray voltage was 2300 V. The FAIMS compensate voltages (CVs) were set to − 45 and − 65 V. Precursors and fragments were analyzed using an Orbitrap detector. The full MS scan resolution was set to 60,000 for a scan range of 400–1200 m/z. The MS/MS scan fixed the first mass to 110 m/z at a resolution of 15,000 with TurboTMT set to off. Up to 25 of the most abundant precursors were then selected for further MS/MS analyses with a 20-s dynamic exclusion. HCD fragmentation was performed at a normalized collision energy (NCE) of 27%. The automatic gain control (AGC) target was set to 100%, with an intensity threshold of 50,000 ions/s and a maximum injection time set to Auto. The resulting MS/MS data were processed using the PD search engine (v.2.4). Trypsin/P was specified as the cleavage enzyme, allowing up to four missing cleavages. In the first search, the mass tolerance for precursor ions was 10 ppm, and the mass tolerance for fragment ions was set as 0.02 Da. Carbamidomethyl on Cys was specified as a fixed modification, and lactylation was specified as a variable modification. The FDR was adjusted to < 1%. The minimum peptide ion scoring requirement was set to above 0, with the identification confidence set to low.

### Structural modeling, lactylation modification, molecular dynamics simulations, and protein–protein Docking of HIF-1α and VHL

To investigate the structural basis of K644 or K12 lactylation in HIF-1α and its potential effects on the VHL interaction, we performed structural prediction, in silico posttranslational modification, molecular dynamics (MD) simulations, and docking analyses. The three-dimensional structures of wild-type (WT) HIF-1α, K644- or K12-lactylated HIF-1α, and the K644R mutant were predicted using AlphaFold3 based on the mouse (UniProt ID: Q61221) and human (UniProt ID: Q16665) HIF-1α sequences retrieved from the UniProt database (https://www.uniprot.org/). Lactylation at Lys644 (in mice) or Lys12 (in humans) was introduced using PyMOL (v3.1.3), Gaussian 16, and GaussView 6.0. Nonstandard residue topology files for lactyl-lysine were generated with Sobtop (v1.0 dev5). For molecular dynamics simulations, the modified protein structures were subjected to preparation and simulation using GROMACS 2024.3. Initial structural files (.gro) were generated using the pdb2gmx tool, during which missing atoms were added and terminal residue defects were ignored. The topology files (.top) were manually edited to incorporate force-field parameters for the added bonds, angles, and dihedrals. A cubic simulation box was created around the protein using editconf, and the system was solvated via solvation with explicit water molecules. Counterions were added with genion to neutralize the system. Energy minimization was performed using grompp and mdrun, followed by two equilibration phases: an NVT (constant number, volume, and temperature) ensemble at 300 K and an NPT (constant number, pressure, and temperature) ensemble at 300 K and 1 bar (compressibility = 4.5e-5 bar⁻¹). A 10-ns production MD simulation was subsequently conducted to analyze the structural dynamics. Root-mean-square deviation (RMSD) values were calculated to assess structural changes, where a lower RMSD indicates less deviation from the starting structure. Distances between key amino acids (e.g., between K644 and P402 or P577) were measured using the carbon atom bound to the carboxyl oxygen as a reference and visualized with PyMOL. For protein–protein docking, the predicted structures of mouse and human HIF-1α, as well as human VHL (UniProt ID: P40338), were downloaded in PDB format. Docking was performed using ClusPro 2.0 (https://cluspro.bu.edu/). The docking poses were visualized and analyzed in PyMOL to assess the potential steric or allosteric effects of lysine modifications on the HIF-1α–VHL interaction.

### ChIP assay

ChIP assays were performed using a Pierce Agarose ChIP Kit (Cat. No. 26156, Thermo Fisher Scientific) according to the manufacturer’s protocols. Briefly, cells were crosslinked with 1% formaldehyde at room temperature for 10 min. Crosslinking was quenched by adding glycine to a final concentration of 0.125 M. After washing with cold PBS containing protease inhibitors, the cells were lysed in 1 mL of SDS lysis buffer (1% SDS, 10 mM EDTA, 50 mM Tris, pH 8.1). Chromatin was then digested with micrococcal nuclease to obtain 100–700 bp fragments. For each ChIP reaction, 10% of the chromatin was stored as the input, and the rest was processed for IP by incubation with RNA polymerase II (as a positive control), rabbit IgG (as a negative control), or MYC antibody at 4 °C overnight. After degrading the proteins in the precipitated complexes with proteinase K, the ChIP-purified DNA was isolated and used as a template for qRT–PCR with the primers indicated in Supplementary Table S3. The qRT–PCR products were then separated via electrophoresis on a 2% agarose gel. The amount of immunoprecipitated DNA in each experiment is represented as a signal relative to the amount of input chromatin.

### Statistical analysis

Statistical analyses were performed using SPSS software version 20.0 (SPSS). The data are presented as the means ± standard errors of the means (SEMs). All the experiments were repeated at least three times. Group differences were analyzed using one-way analysis of variance (ANOVA), followed by the least significant difference (LSD) post hoc test. *P* > 0.05 was considered not significant (NS); *P < 0.05, **P < 0.01, and ***P < 0.001 were considered statistically significant.

## Results

### Hypoxia-induced lactate production facilitates the accumulation of the HIF-1α protein

**Fig. 1:**
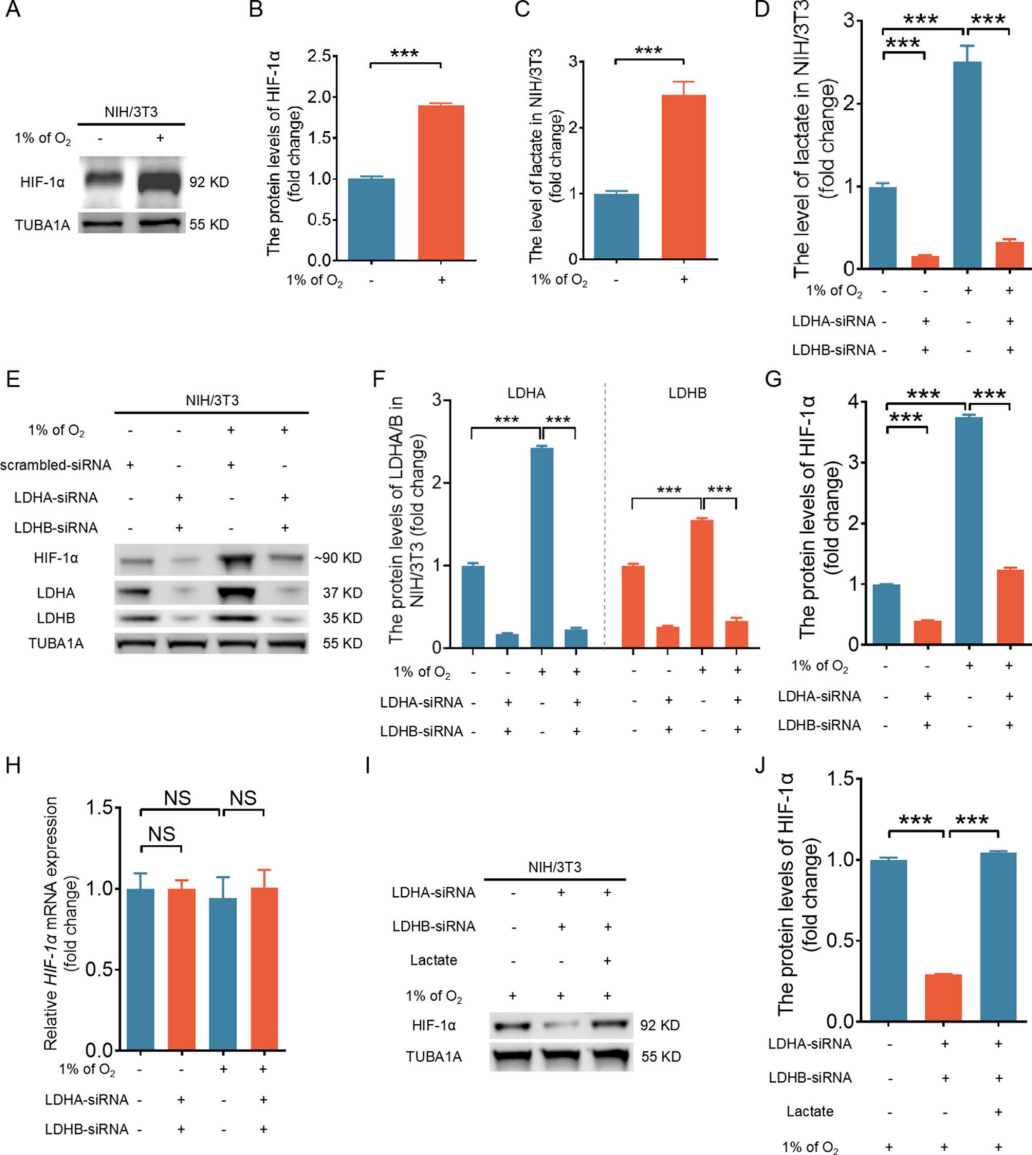
Lactate contributes to the accumulation of HIF-1α protein in hypoxic NIH/3T3 cells. A–C, NIH/3T3 cells were cultured under normoxic (21% O_2_) or hypoxic (1% O_2_) conditions for 6 h and then collected to determine the levels of HIF-1α (**A** and **B**) and lactate (**C**). D–H, NIH/3T3 cells transfected with LDHA/LDHB siRNAs or scramble control siRNA for 24 h were cultured for an additional 6 h under normoxic (21% O_2_) or hypoxic (1% O_2_) conditions and then collected to determine the lactate (**D**) and protein levels of HIF-1α, LDHA, and LDHB (**E**) and the HIF-1α mRNA levels (**H**). Quantification of the immunoblot bands is shown in (**F**) and (**G**). I and J, NIH/3T3 cells transfected with LDHA/LDHB siRNAs or scramble control siRNA for 24 h were treated with or without 1 mM sodium lactate and cultured under hypoxic (1% O_2_) conditions for an additional 6 h. The HIF-1α protein level was determined by western blot (**I**) and quantified (**J**). The data are presented as the means ± s.e.m.s (*n* = 3). ***P < 0.001

Lactate is a byproduct of cellular anaerobic metabolism. Previous studies have demonstrated that lactate can improve the stability of the HIF-1α protein [9–11]. However, it remains unclear whether lactate is required for hypoxia-induced HIF-1α stabilization. In primary porcine granulosa cells (GGs) and NIH/3T3 cells cultured under hypoxia (1% O_2_), we observed that HIF-1α accumulation (Fig. 1A and B, S1A, and S1B) was accompanied by the upregulation of cellular lactate (Fig. 1C and S1C). To investigate the association between HIF-1α accumulation and lactate production, we knocked down LDHA and LDHB to inhibit lactate generation in GGs (Fig. S1D) and NIH/3T3 cells (Fig. 1D). We found that inhibiting lactate generation abrogated the accumulation of HIF-1α protein under both hypoxic and normoxic conditions (Fig. 1E-G, S1E-G) without affecting HIF-1α mRNA levels (Fig. 1H). To confirm whether hypoxia depends on lactate to regulate HIF-1α accumulation, we added lactate after LDHA and LDHB were knocked down in GGs and NIH/3T3 cells. Lactate treatment restored hypoxia-induced HIF-1α accumulation despite the depletion of LDHA and LDHB (Fig. 1I and J, S1H and S1I). Therefore, these data suggest that lactate production is involved in the hypoxic upregulation of HIF-1α protein levels.

### Lactate inhibits the ubiquitination and proteasomal degradation of the HIF-1α protein

**Fig. 2:**
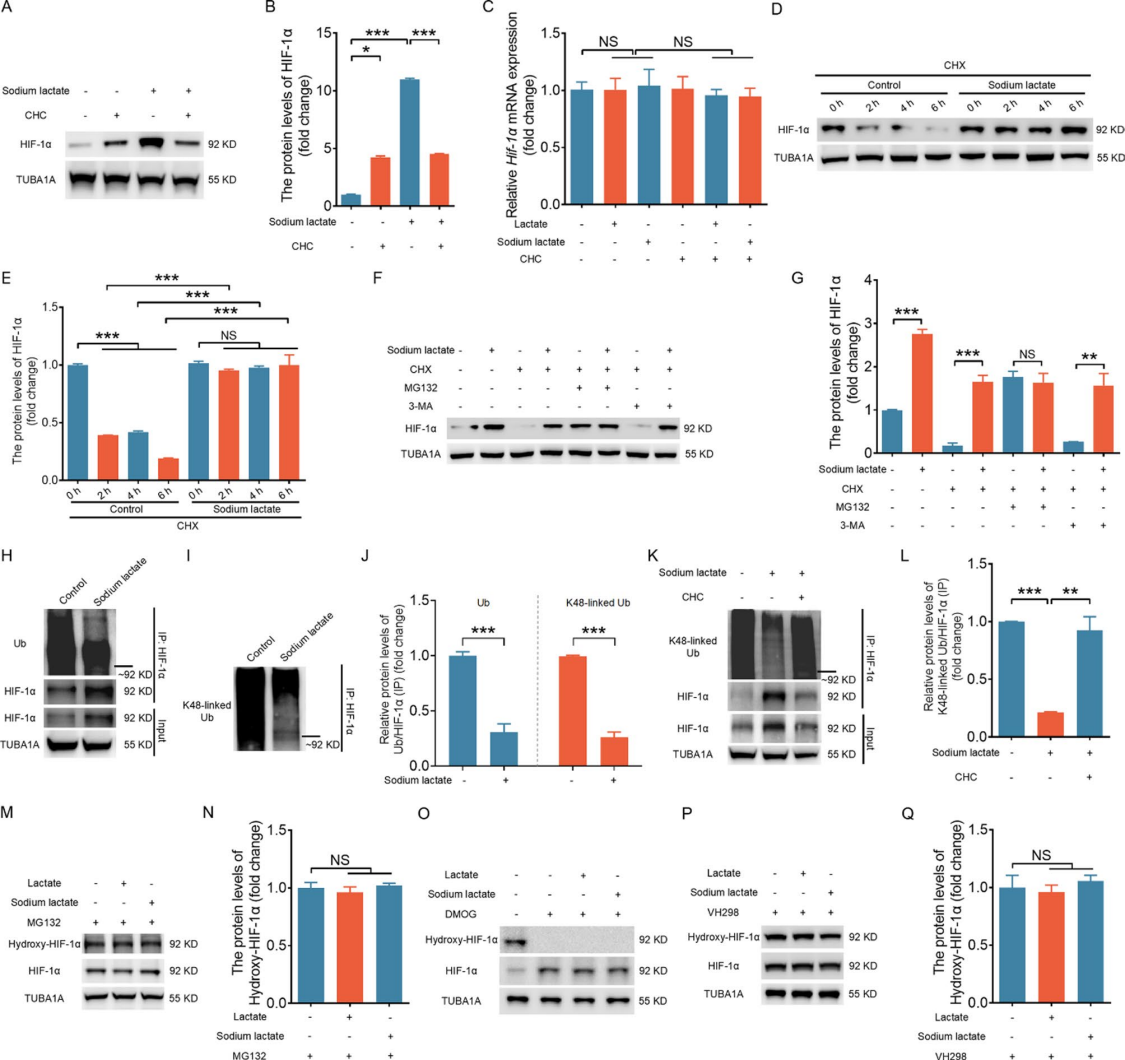
Lactate prevents HIF-1α degradation by inhibiting the ubiquitin–proteasome pathway. A–C, NIH/3T3 cells pretreated with or without 3 mM CHC (monocarboxylate transporter inhibitor, MCT) for 2 h were cultured with or without 1 mM sodium lactate for an additional 6 h and then collected to determine the protein (**A** and **B**) and mRNA expression levels of HIF-1α (**C**). D and E, NIH/3T3 cells pretreated with or without 50 µM cycloheximide were cultured with 1 mM sodium lactate for 0 h, 2 h, 4 h, or 6 h. HIF-1α protein levels were determined by western blotting (**D**) and quantified (**E**). F and G, NIH/3T3 cells were pretreated for 2 h with 50 µM cycloheximide alone, 50 µM cycloheximide, 10 µM MG132, or 5 mM 3-MA, followed by incubation with 1 mM sodium lactate for 6 h. HIF-1α protein levels were then determined by western blotting (**F**) and quantified (**G**). H–J, NIH/3T3 cells were cultured with 1 mM sodium lactate for 6 h under normoxic (21% O_2_) conditions. Immunoprecipitation (IP) was then performed to measure the ubiquitination (**H**) and K48-linked ubiquitination (**I**) levels of the HIF-1α protein, which were quantified (**J**). K and L, NIH/3T3 cells pretreated with 3 mM CHC for 2 h were cultured with 1 mM sodium lactate for 6 h. The K48-linked ubiquitination level of the HIF-1α protein was determined by IP (**K**) and quantified (**L**). M–Q, NIH/3T3 cells pretreated with 10 µM MG132 (M and N), 100 µM DMOG (**O**) or 100 µM VH298 (P and Q) for 2 h were cultured with 10 mM lactate or 1 mM sodium lactate for 6 h. The protein level of hydroxylated HIF-1α was determined by western blot (**M, O** and **P**) and quantified (**N** and **Q**). The data are presented as the means ± s.e.m.s (*n* = 3). *P < 0.05, **P < 0.01, ***P < 0.001; NS, not significant (*P* > 0.05)

To rule out potential nonspecific effects of hypoxia, we added lactate or sodium lactate to NIH/3T3 cells cultured under normoxia. We observed a significant increase in HIF-1α protein levels, which was blocked after treatment with the MCT inhibitors CHC and AZD3965 (Figs. 2 A, 2B, and S2A-H). To elucidate the mechanisms responsible for the lactate-induced increase in HIF-1α protein levels, we initiated our investigation by examining HIF-1α expression at the transcript level via qRT–PCR. As shown in Fig. 2C, we observed no significant changes in HIF-1α mRNA levels before and after lactate/sodium lactate administration. These findings suggest that lactate likely increases HIF-1α protein levels by inhibiting translation or facilitating protein degradation. We employed cycloheximide (CHX), a standard protein synthesis inhibitor used to assess protein degradation, to suppress the de novo synthesis of the HIF-1α protein. Intriguingly, even under conditions of suppressed protein synthesis, sodium lactate continued to promote HIF-1α protein expression (Fig. 2D and E). Moreover, knockdown of LDHA and LDHB significantly attenuated hypoxia-induced HIF-1α accumulation under CHX treatment (Fig. S3). These findings suggest that lactate/sodium lactate increases HIF-1α protein levels primarily by inhibiting its degradation. As is widely recognized, intracellular proteins typically undergo degradation through the autophagic–lysosomal pathway (ALP) and the ubiquitin–proteasome system (UPS) [16]. To further explore the pathway by which lactate/sodium lactate inhibits HIF-1α protein degradation, we treated cells pretreated with cycloheximide with either the autophagy inhibitor 3-MA or the proteasomal inhibitor MG132. As shown in Fig. 2F and G, lactate/sodium lactate-induced accumulation of the HIF-1α protein persisted with the addition of 3-MA but disappeared in the presence of MG132, indicating that the UPS pathway contributes to the inhibitory effects of lactate/sodium on HIF-1α protein degradation. IP revealed that sodium lactate-induced HIF-1α accumulation was accompanied by decreased levels of ubiquitinated HIF-1α protein (Fig. 2H and J and Fig. S4), especially K48-linked polyubiquitination of the HIF-1α protein (Fig. 2I and J), which was reversed by treatment with the MCT inhibitor CHC (Fig. 2K and L).

We next investigated how lactate inhibits the ubiquitination of the HIF-1α protein. Given that HIF-1α is hydroxylated on specific proline residues for subsequent ubiquitination [3], we examined whether lactate might affect the hydroxylation of the HIF-1α protein. As shown in Fig. 2M and Q, in cells pretreated with MG132 (which inhibits HIF-1α protein degradation) (Fig. 2M and N), DMOG (which inhibits prolyl hydroxylation of HIF-1α) (Fig. 2O), or VH298 (a VHL inhibitor) (Fig. 2P and Q), sodium lactate did not affect the hydroxylation levels of the HIF-1α protein. These results indicate that lactate can inhibit HIF-1α protein degradation through alternative pathways independent of hydroxylation.

### Lactate-induced lactylation of HIF-1α is accompanied by decreased levels of HIF-1α ubiquitination and impaired binding affinity between HIF-1α and VHL

**Fig. 3:**
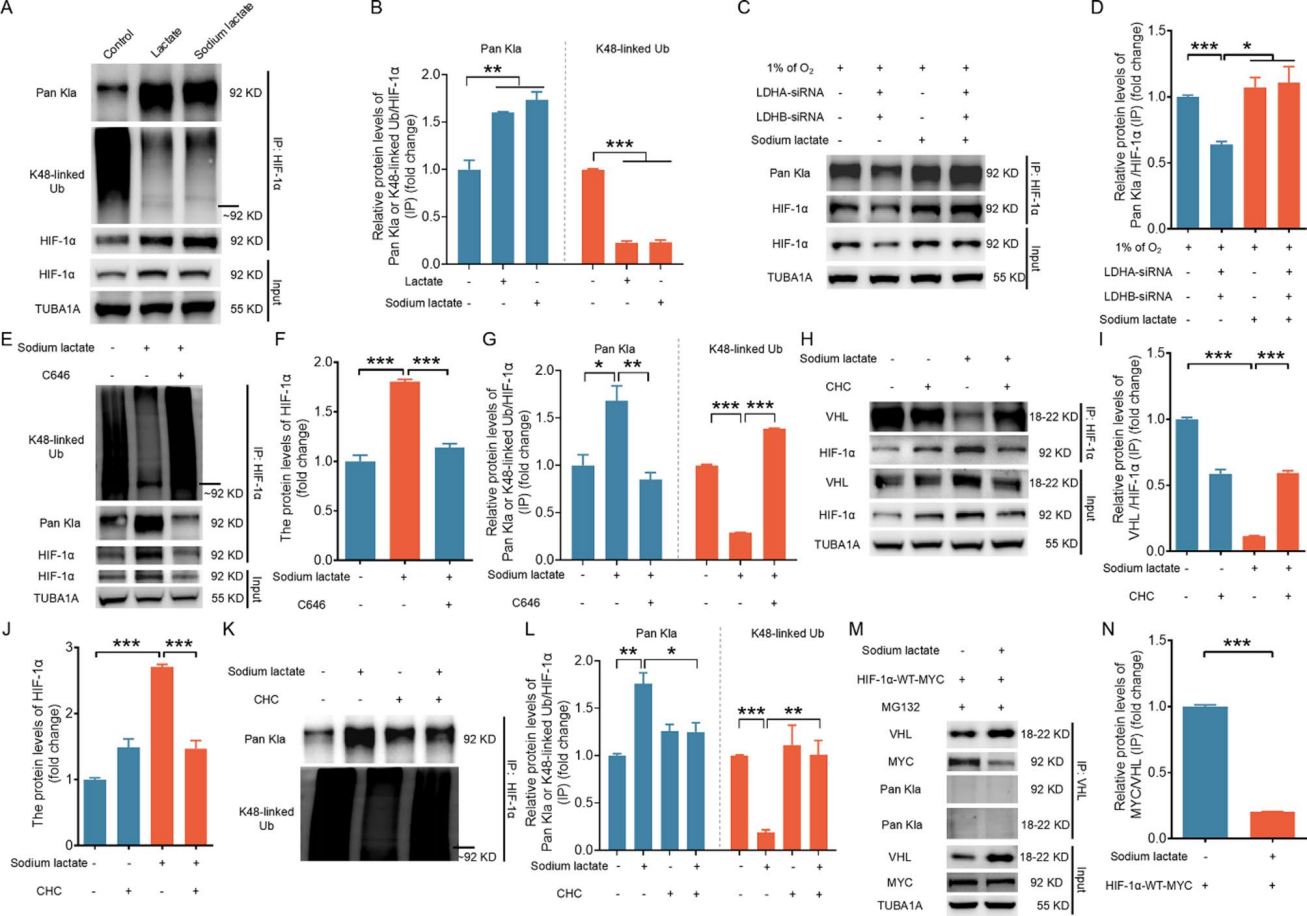
Lactate-induced HIF-1α lactylation is associated with decreased levels of HIF-1α ubiquitination and compromised binding affinity between HIF-1α and VHL. A and B, NIH/3T3 cells were cultured with 10 mM lactate or 1 mM sodium lactate for 6 h. The lactylation and K48-linked ubiquitination levels of the HIF-1α protein were determined by IP (**A**) and quantified (**B**). C and D, NIH/3T3 cells transfected with LDHA/LDHB siRNAs or scramble control siRNA for 24 h were treated with or without 1 mM sodium lactate and cultured under hypoxic (1% O_2_) conditions for an additional 6 h. The HIF-1α protein level was measured by western blotting (**C**). The interaction between HIF-1α and pan-Kla was determined by immunoprecipitation (IP) (**C**) and quantified (**D**). E–G, NIH/3T3 cells pretreated with 10 µM C646 for 2 h were cultured with 1 mM sodium lactate for 6 h. The lactylation and K48-linked ubiquitination levels of the HIF-1α protein were determined by IP (**E**) and quantified (**F** and **G**). H–L, NIH/3T3 cells pretreated with 3 mM CHC for 2 h were cultured with 1 mM sodium lactate for 6 h. The protein levels of HIF-1α were measured by western blotting (**H**). IP was performed to analyze the interaction between HIF-1α and VHL (**H**), as well as the lactylation and K48-linked ubiquitination levels of the HIF-1α protein (**K**), and the results were quantified (**I**, **J** and **L**). M and N, NIH/3T3 cells pretreated with 10 µM MG132 for 2 h were transfected with HIF-1α-WT-MYC and then cultured with or without 1 mM sodium lactate for 6 h. The protein levels of VHL and MYC were measured by western blot (**M**), and IP was performed to analyze the interaction between VHL and MYC or pan-Kla (**M**), and the results were quantified (**N**). The data are presented as the means ± s.e.m.s (*n* = 3). *P < 0.05, **P < 0.01, ***P < 0.001

Lactylation, which is regulated by lactate, has recently been identified as a novel contributor to protein functions [17]. As shown in Fig. S5, blocking the activity of P300, the main “writer” enzyme for lactylation with its specific antagonist C646, inhibited sodium lactate-induced accumulation of the HIF-1α protein, suggesting a possible association between HIF-1α protein accumulation and lactylation in response to sodium lactate treatment. Next, we determined whether HIF-1α undergoes lactylation and its contribution to HIF-1α protein accumulation following treatment with sodium lactate. As shown in Fig. 3A and B and S6A-F, lactate and sodium lactate strongly increased Kla levels in the HIF-1α immunocomplex. In addition, lactate/sodium lactate significantly reduced K48-linked polyubiquitination of HIF-1α (Fig. 3A and B). Moreover, the lactylated HIF-1α levels were considerably decreased after LDHA and LDHB were knocked down under hypoxia, whereas repletion with sodium lactate restored hypoxia-induced HIF-1α accumulation and HIF-1α lactylation despite the depletion of LDHA and LDHB (Fig. 3C and D). Importantly, blocking P300 activity with C646 significantly inhibited HIF-1α lactylation while restoring its ubiquitination in NIH/3T3, GC, and 293T cells treated with sodium lactate (Figs. 3E–G and S7A–F). However, C646 failed to repress HIF-1α accumulation and HIF-1α lactylation further when LDHA and LDHB were transcriptionally silenced under hypoxia (Fig. S8A–C). Overall, these results suggest that lactate might act via HIF-1α lactylation to inhibit the ubiquitination and degradation of HIF-1α.

The HIF-1α protein undergoes ubiquitination via recognition by the E3 ubiquitin ligase VHL [2, 3]. These findings increase the possibility that the lactylation of HIF-1α inhibits its ubiquitination by influencing the recognition of VHL. To validate this assumption, we examined the interaction between HIF-1α and VHL following treatment with sodium lactate. As shown in Fig. 3H–L, the lactylation of HIF-1α induced by sodium lactate is associated with impaired binding affinity between HIF-1α and VHL and reduced levels of ubiquitinated HIF-1α. In contrast, upon treatment with CHC to inhibit lactate-induced HIF-1α lactylation, the interaction between VHL and HIF-1α (Fig. 3H-J) and the ubiquitinated HIF-1α level (Fig. 3K and L) were partially restored. Consistent results were obtained in cells transfected with MYC-HIF-1α, which revealed an impaired interaction between VHL and MYC-HIF-1α following sodium lactate treatment (Fig. 3M and N). Notably, IP using the VHL antibody revealed no lactylation modification of the complex of VHL and its associated proteins, suggesting that VHL might bind to only the nonlactylated form of HIF-1α. These results suggest that lactylation of the HIF-1α protein might inhibit the recognition of HIF-1α by VHL, thus preventing its ubiquitination and subsequent degradation.

### Identification of lactylation sites in HIF-1α across species

**Fig. 4:**
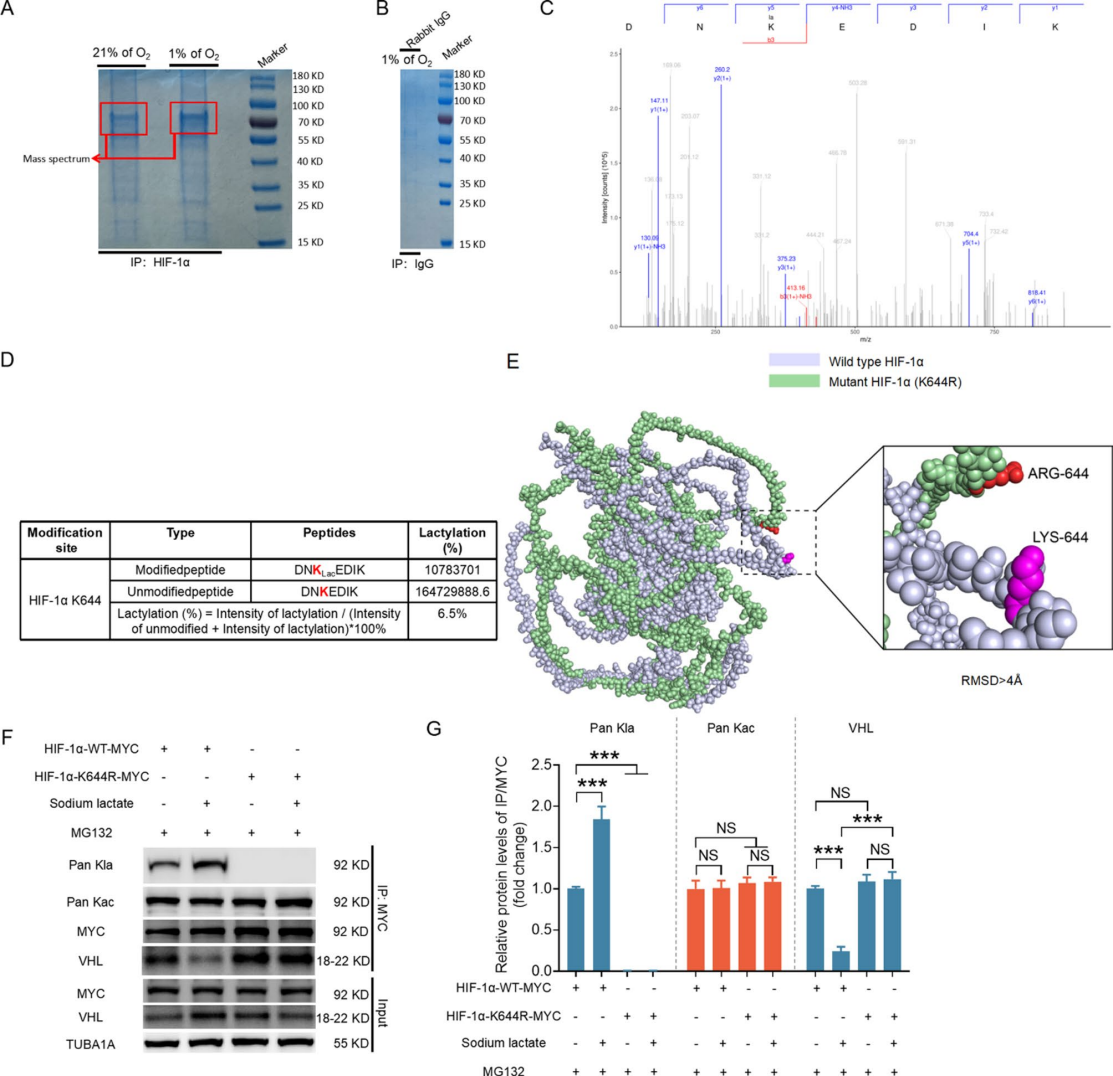
Identification of K644 as a unique lactylation site on HIF-1α. A and B, NIH/3T3 cells cultured under normoxic (21% O_2_) or hypoxic (1% O_2_) conditions for 6 h were collected for IP reactions with HIF-1α (**A**) or rabbit IgG (**B**) antibodies. The IP samples were then subjected to polyacrylamide gel electrophoresis, followed by Coomassie brilliant blue staining. **C**, K644 was identified as the lactylation site of HIF-1α via mass spectrometry analysis. **D**, Calculation of the proportion of lactylation modifications at K644 of HIF-1α. **E**, Comparison of the wild-type and K644R mutant HIF-1α protein structures using AlphaFold3; the stick structures of K644 and R644 are marked. The root mean square deviation (RMSD) value quantifies structural deviations, with lower RMSD values indicating minimal impact on the spatial structure of the protein. **F–G**, NIH/3T3 cells were transfected with vectors encoding MYC-tagged wild-type HIF-1α or the K644R mutant for 24 h. After transfection, the cells were pretreated with 10 µM MG132 for 2 h, followed by incubation with 1 mM sodium lactate for 6 h. Western blotting was performed to measure MYC and VHL protein levels (**F**), and IP was used to assess the interaction between MYC and VHL, as well as the lactylation and acetylation levels of MYC-tagged HIF-1α (**F**). The quantified data are shown in (**G**). The data are presented as the means ± s.e.m.s (*n* = 3). *P < 0.05, **P < 0.01, ***P < 0.001; NS, not significant (*P* > 0.05)

To further elucidate how lactylation of HIF-1α impedes the recognition process by VHL, we attempted to identify the lactylation sites of the HIF-1α protein via mass spectrometry. NIH/3T3 cells were cultured under either normoxic or hypoxic conditions for 6 h, followed by IP reactions with either the HIF-1α antibody (Fig. 4A) or the rabbit IgG antibody (Fig. 4B). The precipitates were subsequently denatured, subjected to polyacrylamide gel electrophoresis, and stained with Coomassie brilliant blue. As shown in Fig. 4A, gel sections corresponding to the molecular weight of HIF-1α were excised based on protein markers. After protein extraction and trypsin digestion, liquid chromatography–tandem mass spectrometry (LC–MS/MS) analysis was performed, and the secondary mass spectrometry data were searched using Proteome Discoverer 2.4. Finally, we identified a single lactylated lysine residue in HIF-1α at position K644 (Fig. 4C). Mass spectrometry analysis revealed no lactylation of HIF-1α in the normoxia group. In contrast, lactylation at K644 of HIF-1α was observed in the hypoxia group, accounting for 6.5% of the total HIF-1α (Fig. 4D). To further investigate the role of K644 in HIF-1α lactylation, we introduced a K644R mutation to mimic the delactylated state of the protein. AlphaFold3 analysis revealed that the K644R mutation altered the protein structure of HIF-1α (Fig. 4E). To rule out interference caused by differences in HIF-1α protein levels, we used MG132 to inhibit HIF-1α degradation before we transfected cells with wild-type HIF-1α (HIF-1α-WT-MYC) and the K644R mutant (HIF-1α-K644R-MYC). As shown in Fig. 4F–I, both the MYC-tagged HIF-1α WT and HIF-1α K644R mutant groups exhibited successful overexpression of MYC-HIF-1α. Importantly, in cells subjected to sodium lactate or hypoxia, HIF-1α lactylation was completely abrogated in the K644R mutant group compared with the WT group. In addition, the K644 mutation did not affect the acetylation levels of HIF-1α. These data suggest that K644R is a unique lactylation site in HIF-1α. To test whether HIF-1α K644 lactylation (K644la) affects its binding with VHL, we first generated three-dimensional structural models of wild-type (WT) HIF-1α, K644R mutant HIF-1α, and VHL proteins using AlphaFold 3 based on structural information obtained from the UniProt database (HIF-1α: Q61221; VHL: P40338) (Fig. S9A and S9B). Molecular docking between the HIF-1α and VHL structures was performed using ClusPro 2.0 (Fig. S9A and S9B). The docking model with the highest binding affinity (lowest energy score) was selected. The results revealed that the energy scores for the WT HIF-1α-VHL and K644R mutant HIF-1α-VHL complexes were − 1297.253 and − 1353.354, respectively, indicating that the K644R mutation itself does not significantly affect the interaction between HIF-1α and VHL. To further validate this finding, we performed anti-MYC IP after transfecting MYC-tagged HIF-1α and MYC-tagged K644R mutant HIF-1α into cells treated with sodium lactate or subjected to hypoxia. Under basal conditions (Fig. 4F-G, Fig. S10A and zzS10B), the K644R mutation did not disrupt the HIF-1α-VHL interaction, validating the computational predictions from our molecular docking analysis. In contrast, under sodium lactate treatment or hypoxic stimulation, this mutation rescued the lactylation-induced impairment of the HIF-1α-VHL interaction (Fig. 4F-G, Fig. S10A and S10B), demonstrating the essential role of K644-specific lactylation in blocking VHL recognition.

Using modification-specific mass spectrometry, we identified K644 as the only lactylation site in mouse HIF-1α. To explore whether lactylation-mediated inhibition of HIF-1α degradation is a conserved mechanism, we compared the amino acid sequences of HIF-1α across humans, pigs, and mice (Fig. S11A). Interestingly, the lysine at position 644 is present only in mouse HIF-1α, whereas the corresponding residue is methionine in both humans and pigs. Currently, there are no reports indicating that methionine undergoes lactylation. Furthermore, experiments conducted with human and porcine cells (Fig. S7) confirmed that the lactylation of HIF-1α inhibits its K48-linked ubiquitination. However, in pig human/porcine HIF-1α, the residue corresponding to mouse K644 is a methionine in both species, indicating that lactylation must occur elsewhere. Because K644 is the only site modified in mice—and none of the lysines conserved across mice, pigs, and humans appear to be lactylated—this suggests that pig and human lactylation targets nonconserved lysines. Sequence alignment revealed four such candidate sites: K12, K156, K649, and K759 (Fig. S11A). We thus generated MYC-tagged K-to-R mutant constructs for each site and transfected them into 293T cells. Following MG132 pretreatment and sodium lactate incubation, we performed MYC immunoprecipitation (IP). As shown in Fig. S11B and S11C, only the K12R mutation completely abolished lactylation induced by sodium lactate, similar to the K644R mutation in mice. In contrast, the K156R, K649R, and K759R mutations did not affect lactylation. Moreover, only the K12R mutation significantly enhanced the K48-linked ubiquitination of HIF-1α and promoted its interaction with VHL, whereas the other mutations had no effect. Structural modeling with AlphaFold3 revealed that the K12R mutation alone produces negligible changes in the overall fold of HIF-1α (Fig. S11D), whereas installation of a lactyl group at K12 triggers pronounced local rearrangements (Fig. S11E). In the unmodified and K12R models, the distances from residue 12 to the hydroxylation sites P402 and P564 essentially remained unchanged (Fig. S11F). In contrast, K12 lactylation shortened both distances (Fig. S11G), providing a plausible allosteric route by which this distal PTM may influence VHL recognition. To further validate K12 as a functional lactylation site in porcine HIF-1α, we generated MYC-tagged wild-type and K12R mutant constructs and transfected them into MG132-pretreated porcine granulosa cells (GCs), followed by sodium lactate treatment. Consistent with the results in 293T cells, the K12R mutation in porcine HIF-1α inhibited lactylation while promoting K48-linked ubiquitination and VHL binding (Fig. S11H and S11I). qRT‒PCR analysis revealed that sodium lactate treatment markedly upregulated the mRNA levels of the canonical HIF-1α targets *Vegfa* and *Glut1*, whereas cells expressing the K12R mutant presented significantly blunted induction of these genes under identical conditions (Fig. S11J). These results suggest that lactylation at K12 enhances the transcriptional activity of HIF-1α. These findings demonstrate that K12 serves as a key lactylation site in human and porcine HIF-1α, playing a crucial role in preventing ubiquitin-mediated degradation.

### HIF-1α K644la inhibits the ubiquitination and degradation of the HIF-1α protein

**Fig. 5:**
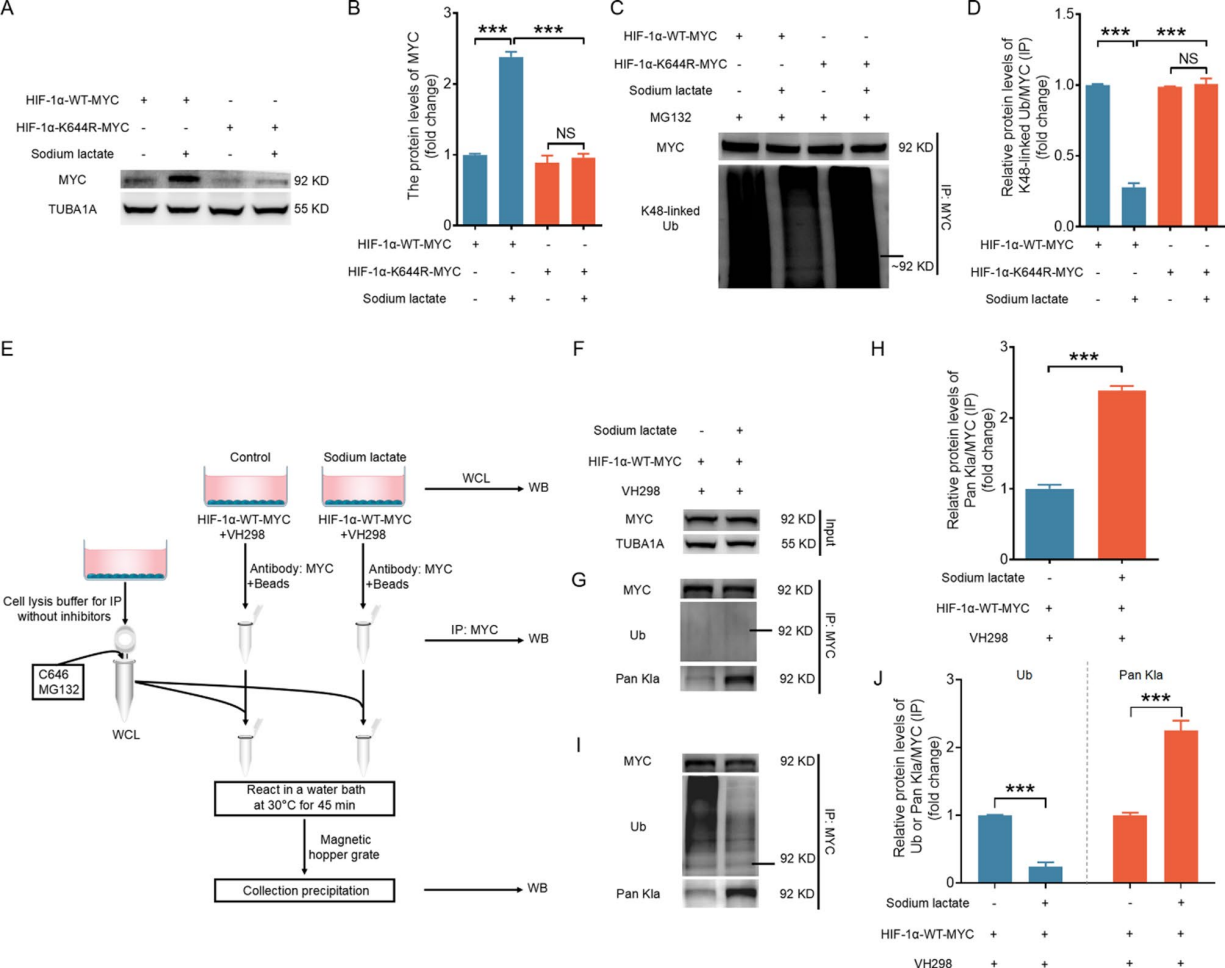
Lactylation at K644 impedes the ubiquitination and degradation of the HIF-1α protein. A–D, NIH/3T3 cells pretreated with or without 10 µM MG132 for 2 h were transfected with the MYC-tagged wild-type HIF-1α vector or K644R mutant vector for 24 h, followed by incubation with 1 mM sodium lactate for 6 h. The protein level of MYC was measured by western blot (**A**) and quantified (**B**). IP was then performed to determine the K48-linked ubiquitination level of the MYC protein (**C**), and the results were quantified (**D**). E–J, Schematic illustrating the lactylated HIF-1α-VHL reaction system depicted in (**E**). Briefly, NIH/3T3 cells transfected with the MYC-tagged wild-type HIF-1α vector for 12 h were treated with 100 µM VH298 (an inhibitor of VHL) for 12 h and then cultured with 1 mM sodium lactate for 6 h. Cells were collected to measure the MYC levels by western blotting (**F**) or subjected to IP in which the MYC antibody was used to determine the lactylation or ubiquitination levels of the MYC protein (**G**), and the results were quantified (**H**). I and J, After IP with an anti-MYC antibody, the precipitates were incubated with whole-cell lysate containing 10 µM C646 and 10 µM MG132 in a 30 °C water bath for 45 min in vitro. The precipitates were subsequently collected to determine the lactylation or ubiquitination levels of the MYC protein (**I**), and the results were quantified (**J**). The data are presented as the means ± s.e.m.s (*n* = 3). *P < 0.05, **P < 0.01, ***P < 0.001; NS, not significant (*P* > 0.05)

To investigate whether HIF-1α lactylation directly regulates its ubiquitination and degradation, we transfected NIH/3T3 cells with MYC-HIF-1α or the MYC-HIF-1α K644R mutant. Figures 5A–D show that the K644R mutation could impede sodium lactate-induced accumulation of the HIF-1α protein (Fig. 5A and B) and block the inhibitory effects of sodium lactate on HIF-1α ubiquitination (Fig. 5C and D). However, the K644R mutation could not completely inhibit HIF-1α accumulation and failed to fully restore HIF-1α ubiquitination under hypoxic conditions (Fig. S12A-D). This might be attributed to the inhibition of HIF-1α hydroxylation by hypoxia. Next, we established a reaction system consisting of two parts: (1) bead-bound MYC-tagged HIF-1α with/without lactylation induced by sodium lactate treatment and (2) cell lysates containing VHL (Fig. 5E). As depicted in Fig. 5F–J, MYC-tagged HIF-1α with an elevated level of lactylation demonstrated a decreased level of ubiquitination after coincubation with cell lysates containing VHL, further confirming that HIF-1α lactylation could inhibit HIF-1α ubiquitination. Collectively, these data show that HIF-1α K644la inhibits the ubiquitination and degradation of the HIF-1α protein.

### The presence of K644la in hydroxylated HIF-1α inhibits its ubiquitination and degradation by preventing VHL recognition

**Fig. 6:**
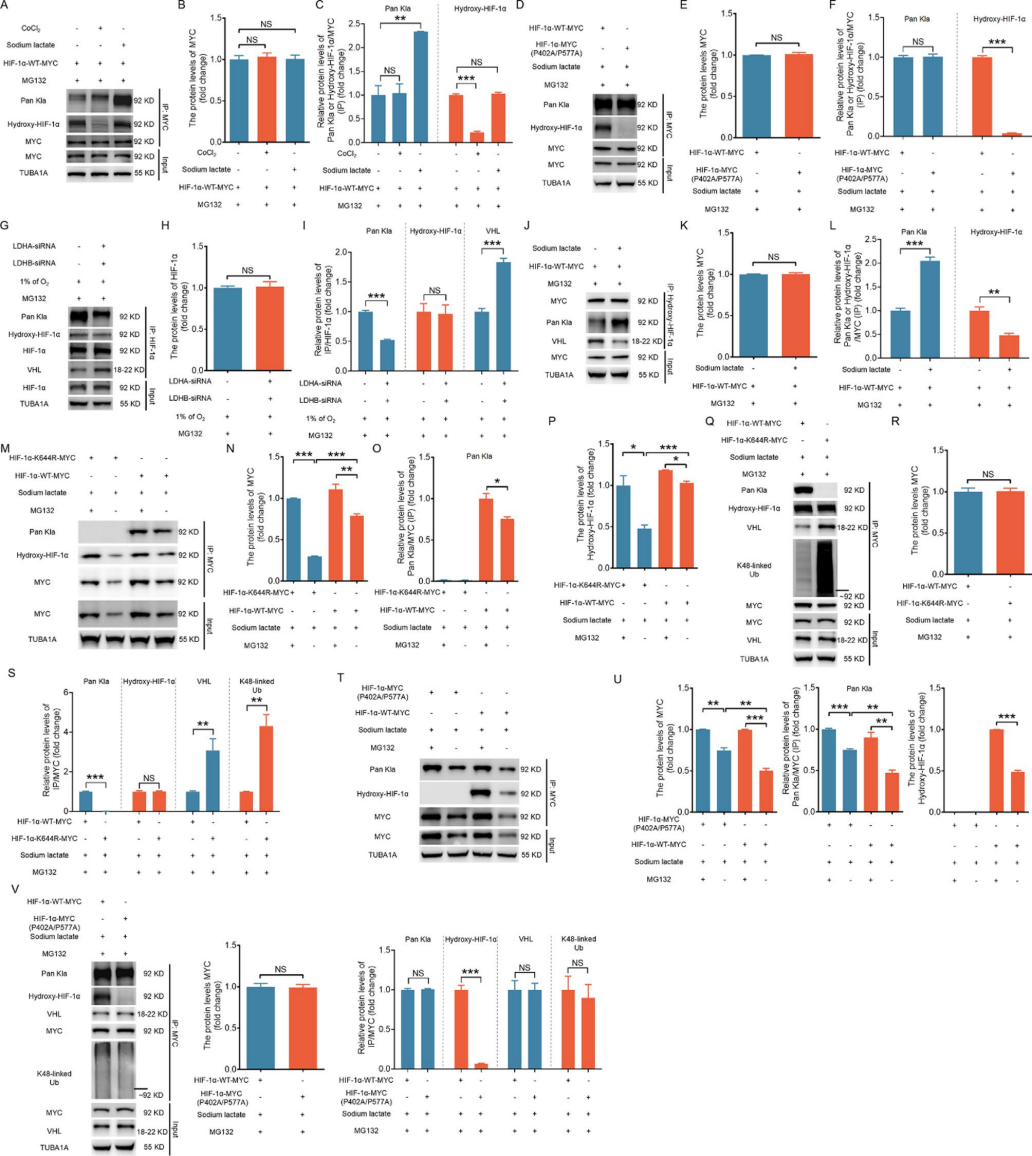
Lactylation of hydroxylated HIF-1α prevents its VHL-dependent ubiquitination and degradation. A–C, NIH/3T3 cells pretreated with 10 µM MG132 for 2 h were transfected with the MYC-tagged wild-type HIF-1α vector for 24 h and then cultured with 100 µM CoCl_2_ or 1 mM sodium lactate for 6 h. IP was performed to determine the lactylation and hydroxylation levels of the MYC protein (**A**), and the results were quantified (**B and C**). **D–F**, NIH/3T3 cells pretreated with 10 µM MG132 for 2 h were transfected with HIF-1α-WT-MYC or HIF-1α-MYC (P402A/P577A) and then cultured with 1 mM sodium lactate for 6 h. IP was performed to detect the lactylation and hydroxylation levels of the MYC protein (**D**), and the results were quantified (**E and F**). **G–I**, NIH/3T3 cells pretreated with 10 µM MG132 for 2 h were cotransfected with LDHA and LDHB-siRNAs for 24 h and then cultured under hypoxia (1% O_2_) for 6 h. IP was performed to analyze the interaction between HIF-1α and VHL and the lactylation and hydroxylation levels of the HIF-1α protein (**G**), and the results were quantified (**H and I**). J–L. NIH/3T3 cells pretreated with 10 µM MG132 for 2 h were transfected with the MYC-tagged wild-type HIF-1α vector for 24 h and then cultured with 1 mM sodium lactate for 6 h. IP was performed to analyze the interaction between MYC and hydroxylated HIF-1α or VHL and hydroxylated HIF-1α, as well as the lactylation level of hydroxylated HIF-1α (**J**), and the results were quantified (**K and L**). M and T, NIH/3T3 cells pretreated with or without 10 µM MG132 for 2 h were transfected with HIF-1α expression vectors encoding HIF-1α-WT-MYC, HIF-1α-K644R-MYC, or HIF-1α-MYC (P402A/P577A) for 24 h and then cultured with 1 mM sodium lactate for 6 h. IP was performed to determine the lactylation and hydroxylation levels of the MYC protein, and the results were quantified. Q and V, NIH/3T3 cells pretreated with 10 µM MG132 for 2 h were transfected with HIF-1α expression vectors encoding HIF-1α-WT-MYC, HIF-1α-K644R-MYC, or HIF-1α-MYC (P402A/P577A) for 24 h and then cultured with 1 mM sodium lactate for 6 h. The protein levels of MYC and VHL were measured via western blot, followed by IP analysis of the interaction between MYC and VHL and the lactylation, hydroxylation, and K48-linked ubiquitination levels of the MYC protein and quantification. The data are presented as the means ± s.e.m.s (*n* = 3). *P < 0.05, **P < 0.01, ***P < 0.001; NS, not significant (*P* > 0.05)

It is commonly accepted that the interaction of VHL with HIF-1α primarily occurs via recognition of hydroxylation modifications at proline residues P402 and P564 within the oxygen-dependent degradation domain (ODD) of HIF-1α [3]. Because lactylation of K644 inhibits the interaction between HIF-1α and VHL (Fig. 4F and G, S10A and S10B), K644la may repress the hydroxylation of HIF-1α. To validate this assumption, we transfected NIH/3T3 cells with the HIF-1α-WT-MYC plasmid in the presence of MG132 to inhibit HIF-1α degradation, followed by treatment with either CoCl_2_ or sodium lactate. IP with the MYC antibody revealed that CoCl_2_ considerably inhibited the hydroxylation of MYC-tagged HIF-1α without affecting lactylation, whereas sodium lactate substantially increased the lactylation of MYC-tagged HIF-1α without altering hydroxylation (Fig. 6A–C). Similarly, the P402A/577 mutation of HIF-1α did not change the level of HIF-1α lactylation upon sodium lactate treatment (Fig. 6D–F). Blocking HIF-1α lactylation by knocking down LDHA and LDHB under hypoxia had no evident influence on HIF-1α hydroxylation but promoted binding between HIF-1α and VHL (Fig. 6G–I). In contrast, overexpressing HIF-1α-WT-MYC in the presence of sodium lactate markedly promoted the lactylation of hydroxylated MYC and considerably inhibited the binding between hydroxylated MYC and VHL (Fig. 6J–L). These data suggest that the lactylation modification of HIF-1α does not affect its hydroxylation (and vice versa) but inhibits the binding of hydroxylated HIF-1α to VHL.

Next, we investigated how hydroxylation and lactylation collaborate in modulating the ubiquitination and degradation of the HIF-1α protein. NIH/3T3 cells were transfected with vectors encoding HIF-1α-WT-MYC and HIF-1α-K644R-MYC in the presence of sodium lactate. As shown in Fig. 6M–P, when MG132 was removed, the levels of both forms of HIF-1α decreased, with the K644R variant showing a significantly greater reduction than WT HIF-1α did, indicating that K644la is required for HIF-1α stabilization. By analyzing the structural model of human HIF-1α (UniProt ID: P68040) using AlphaFold3, we found that the spatial distances between K644 and the hydroxylation sites P402 and P577 remained largely unchanged upon the K644R mutation (Fig. S13A). In contrast, lysine lactylation at K644 induced notable conformational alterations in the HIF-1α protein (Fig. S13B), characterized by a decreased distance between K644 and P577 but an increased distance between K644 and P402 (Fig. S13C). These structural changes suggest that lactylation, unlike simple mutation, may allosterically influence the spatial configuration of the ODD domain. Consistently, overexpression of HIF-1α-WT-MYC or HIF-1α-K644R-MYC in cells treated with MG132 and sodium lactate revealed that the K644R mutation enhanced the interaction between MYC and VHL, as well as the K48-linked ubiquitination of HIF-1α (Fig. 6Q–S). Importantly, this mutation did not alter the hydroxylation status of HIF-1α (Fig. 6M and Q), suggesting that the reduced VHL binding observed upon K644 lactylation is not due to impaired prolyl hydroxylation but likely stems from structural reorganization. We also assessed the impact of HIF-1α hydroxylation on HIF-1α stability mediated by lactylation. NIH/3T3 cells were transfected with either HIF-1α-WT-MYC or HIF-1α-MYC (P402A/P577A) plasmids in sodium lactate. Using the MYC antibody, IP revealed that upon MG132 withdrawal, both forms of HIF-1α decreased, with the wild type showing a more significant reduction than the hydroxylation mutant. Similarly, when MG132 was removed, the hydroxylation level of MYC decreased, whereas the lactylation level remained unchanged (Fig. 6T and U). These findings indicate that the degraded form of HIF-1α is hydroxylated. Additionally, upon the overexpression of HIF-1α-WT-MYC or HIF-1α-MYC (P402A/P577A) in cells treated with MG132 and sodium lactate, the P402A/P577A mutation inhibited the hydroxylation of MYC. Nevertheless, this did not affect its lactylation, VHL binding, or level of K48-linked ubiquitination (Fig. 6V). Our data indicate that once HIF-1α is lactylated, hydroxylated HIF-1α fails to bind to VHL, preventing its ubiquitination and subsequent degradation.

### Lactylation enhances HIF-1α transcriptional activity without altering its nuclear localization

**Fig. 7:**
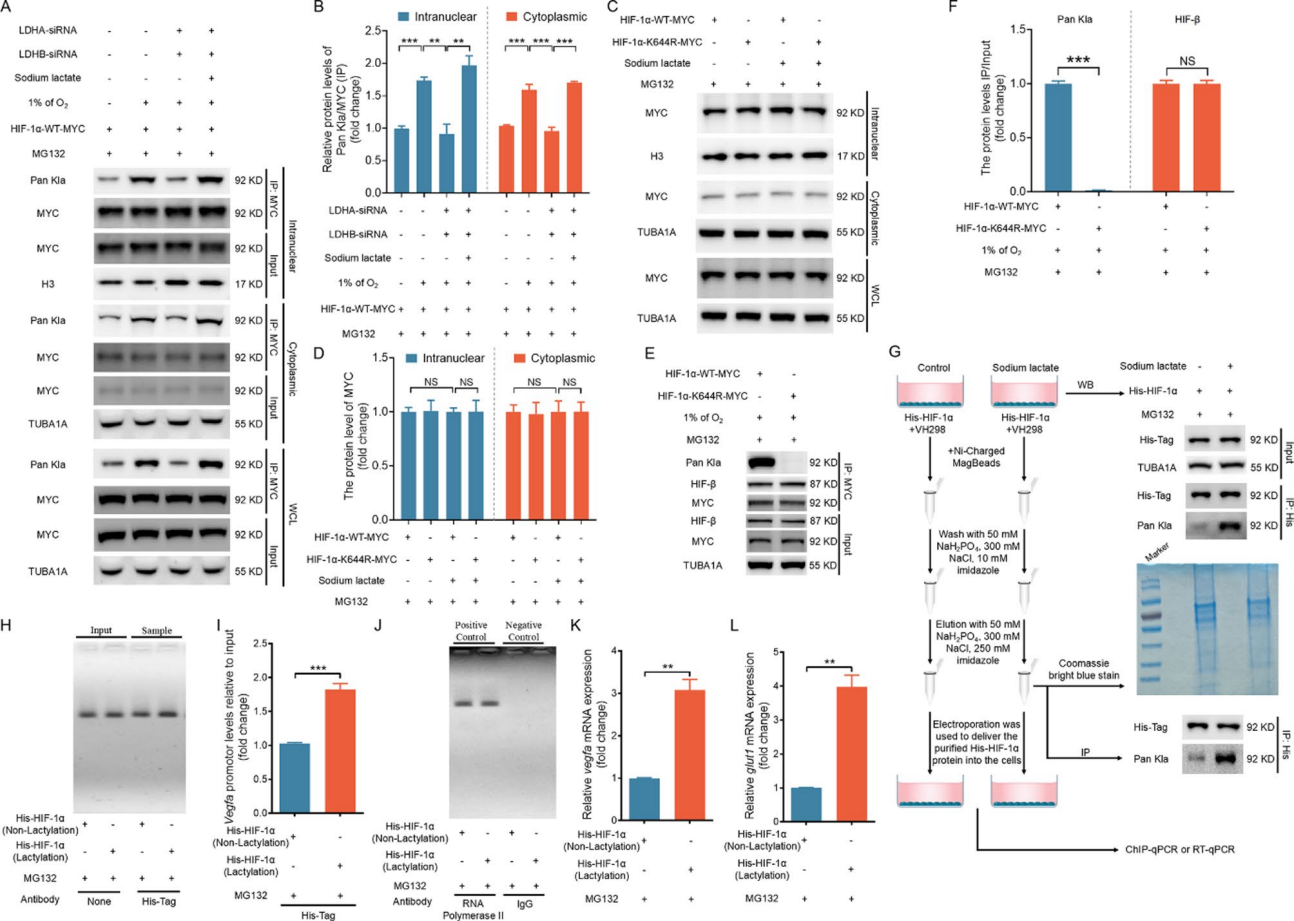
Lactylation of HIF-1α is crucial for its transcriptional activation. A and B, NIH/3T3 cells transfected with LDHA/LDHB siRNAs or scramble control siRNA for 24 h were treated with 10 µM MG132 for 2 h and cultured with or without 1 mM sodium lactate under hypoxic (1% O_2_) conditions for 6 h. The cells were then collected to determine the interaction between MYC and pan-Kla by IP analysis of the cytoplasmic and nuclear fractions (**A**), and the results were quantified (**B**). C and D, NIH/3T3 cells were pretreated with 10 µM MG132 for 2 h and transfected with HIF-1α-WT-MYC or HIF-1α-K644R-MYC for 24 h. Then, the cells were cultured with or without 1 mM sodium lactate for 6 h. WWestern blotting was performed to measure the levels of MYC in the cytoplasmic and nuclear fractions (**C**), and the results were quantified (**D**). E and F, NIH/3T3 cells were pretreated with 10 µM MG132 for 2 h and transfected with HIF-1α-WT-MYC or HIF-1α-K644R-MYC for 24 h. IP was performed to measure the lactylation level of the MYC protein and the interaction between MYC and HIF-β (**E**), and the results were quantified (**F**). G–M: Schematic diagram illustrating the detection of lactylated HIF-1α binding to the *Vegfa* promoter in a chromatin immunoprecipitation (ChIP) assay (**G**). NIH/3T3 cells were pretreated with 100 µM VH298 for 2 h, transfected with a His-HIF-1α vector for 24 h, and then cultured with or without 1 mM sodium lactate for 6 h. The cells were collected to measure the lactylation levels of the His-tagged protein. Following nickel column purification of His-HIF-1α from sodium lactate-treated and untreated cells to obtain lactylated and nonlactylated HIF-1α proteins, the purified proteins were either electroporated into new cells (**H and I**) or added directly to the culture medium without electroporation (**J**), followed by a 6-hour incubation. ChIP analysis was performed to evaluate the binding of His-HIF-1α to the *Vegfa* promoter. DNA from the precipitated complexes was used as a template for qRT–PCR. The resulting qRT–PCR products were analyzed on a 2% agarose gel (**H and J**) and quantified using ImageJ 1.42q software (**I**). The *Gapdh* promoter immunoprecipitated with an RNA polymerase II antibody served as a positive ChIP control, and IgG was used as a negative control (**K**). The mRNA levels of *Vegfa* (**L**) and *Glut1* (**M**) were quantified by qRT–PCR. The data are presented as the means ± s.e.m.s (*n* = 3). *P < 0.05, **P < 0.01, ***P < 0.001; NS, not significant (*P* > 0.05)

As a transcription factor, HIF-1α functions by entering the cell nucleus [18]. For cells cultured under hypoxic conditions, we observed lactylated HIF-1α in both the nucleus and the cytoplasm (Fig. 7A and B). Genetic silencing of LDHA/LDHB suppressed lactylation in both subcellular fractions, whereas exogenous sodium lactate supplementation restored hypoxia-induced lactylation (Fig. 7A and B). However, this modification did not appear to affect the nuclear localization of HIF-1α (Fig. 7A and B). To confirm this, we overexpressed plasmids encoding wild-type (WT) HIF-1α and the K644R HIF-1α mutant in cells treated with sodium lactate. Nuclear‒cytoplasmic fractionation followed by immunoblotting revealed that sodium lactate did not influence the nuclear distribution of either WT or K644R HIF-1α (Fig. 7C and D). To determine whether lactylation modulates HIF-1α transcriptional competence, we performed chromatin immunoprecipitation (ChIP) using cells overexpressing WT or K644R HIF-1α under hypoxia (Fig. 7E and F). Although the K644R mutation abolished lactylation (Fig. 7E-F), it preserved HIF-1α heterodimerization with HIF-1β, confirming the structural integrity (Fig. 7E-F). To directly assess the impact of HIF-1α lactylation on transcriptional regulation, we purified lactylated and nonlactylated HIF-1α proteins through affinity chromatography (Fig. 7G) and reintroduced them into cells. Chromatin immunoprecipitation (ChIP) assays demonstrated that lactylated HIF-1α exhibited a 2.3-fold increase in binding occupancy at the *Vegfa* promoter compared with the nonlactylated form (Fig. 7H-K). Consistent with these chromatin engagement dynamics, qRT–PCR analysis revealed that lactylated HIF-1α elevated the mRNA expression levels of the canonical HIF-1 targets *Vegfa* and *Glut1* in VH298-treated cells, whereas nonlactylated HIF-1α showed minimal transcriptional induction (Fig. 7L and M). These orthogonal approaches conclusively establish that site-specific lactylation serves as a molecular rheostat to potentiate HIF-1α-driven transcriptional programs.

## Discussion

The canonical regulation of HIF-1α protein stability and activity involves a series of posttranslational modifications (PTMs), including hydroxylation, ubiquitination, acetylation, and phosphorylation [19]. Lactylation is a newly discovered protein PTM in which the lactoacyl group is covalently coupled to the protein lysine residue [8]. In this study, we identified lysine lactylation as a conserved regulatory mechanism for HIF-1α stabilization across mammals, albeit through distinct residues: K644 in mice and K12 in humans and pigs. Functionally, this modification impedes the ubiquitination of HIF-1α, preventing its subsequent degradation by the proteasome. Our findings provide an additional layer of the regulatory mechanism governing HIF-1α stability through PTM.

HIF-1α acts as a critical regulator of metabolism, orchestrating glycolysis in response to hypoxic stress by transcriptionally controlling genes that encode glucose transporters and glycolytic enzymes [20]. However, much less is known about whether glycolysis reciprocally affects the stability and activity of the HIF-1α protein. Indeed, the glycolytic metabolite lactate stabilizes HIF-1α [21]; however, the underlying mechanisms remain elusive. Lactylation, which is regulated by lactate, has recently been confirmed to be a critical sensor of metabolic changes [17]. Notably, in prostate cancer cell lines, the accumulation of HIF-1α induced by lactate likely involves lactylation. Our results show that inhibiting lactate uptake with MCT inhibitors suppresses the accumulation of HIF-1α protein, which is accompanied by a decrease in total protein lactylation levels. Luo et al. reported that HIF-1α undergoes lactylation [22]; however, whether this modification impacts HIF-1α stability remains unexplored. Our work provides the first direct evidence that lactylation serves as the molecular link between lactate and HIF-1α stability. Cross-species analyses revealed intriguing differences in lactylation sites: K644 in mice and K12 in humans/pigs. The K–R mutation in this site completely abolished sodium lactate/hypoxia-induced lactylation of HIF-1α but did not affect HIF-1α acetylation levels, supporting the notion that this is a specific lactylation site in HIF-1α. Further studies revealed that inhibition of HIF-1α lactylation by K–R mutation impedes sodium lactate/hypoxia-induced accumulation of the HIF-1α protein and counteracts the inhibitory effect of sodium lactate/hypoxia on the ubiquitin–proteasomal degradation of HIF-1α. This study highlights a novel aspect of lactylation to explain the mechanism underlying the metabolic regulation of HIF-1α stability.

Although our immunoprecipitation and western blot analyses revealed detectable HIF-1α lactylation under normoxic conditions upon lactate treatment (Figs. 3A-B and 4F and G, S10A and S10B), the mass spectrometry data failed to identify K644la under normoxia. This apparent discrepancy likely stems from methodological differences in sensitivity between the two techniques. Mass spectrometry, while providing precise site-specific identification of PTMs, is inherently limited by peptide ionization efficiency and modification stoichiometry. The estimated 6.5% lactylation occupancy at K644 under hypoxia (Fig. 4D) approaches the lower detection threshold of conventional LC‒MS/MS workflows (1–5% for PTM stoichiometry), whereas normoxic lactylation levels may fall below this threshold owing to reduced lactate availability under basal conditions. In contrast, pan-Kla antibody-based detection (e.g., western blot) amplifies signals through enzymatic or chemiluminescent readouts, enabling the detection of low-abundance PTMs that escape MS identification.

The recognition of HIF-1α by the VHL protein is crucial for the ubiquitin-mediated proteasomal degradation of HIF-1α [2, 23]. VHL binds to a region of HIF-1α known as the ODD (aa 401–603) [24]. This domain undergoes certain forms of PTMs that influence VHL recognition. For example, PHDs catalyze the hydroxylation of proline residues (P402 and P564) within the ODD of HIF-1α, promoting its association with VHL [2, 3]. In another scenario, the ARD1 protein specifically acetylates K532 in the ODD of HIF-1α, leading to VHL-dependent HIF-1α protein degradation [25]. Lactylation shares many similarities with acetylation, both chemically and functionally [26], suggesting that lactylation might also affect the recognition of HIF-1α by VHL. To confirm this, we cultured cells under hypoxia to eliminate interference from the PHD–hydroxylation and ARD1–acetylation pathways (both pathways are activated under normoxia and inactivated under hypoxia) on the binding of HIF-1α with VHL. Our data showed that blocking HIF-1α lactylation via the K644R/K12R mutation restored the binding of VHL with HIF-1α. Additional studies have shown that the inhibition of lactylation under hypoxia considerably reduces the accumulation of HIF-1α, reaching levels nearly comparable to those under normoxic conditions. Therefore, these results reveal a key mechanism involving lactylation in the modulation of VHL-mediated degradation of HIF-1α.

It has long been known that HIF-1α binds to VHL via proline hydroxylation under normoxic conditions, leading to the rapid degradation of HIF-1α [3]. Unexpectedly, in cells cultured under normoxia, lactate inhibited HIF-1α degradation by promoting its lactylation. These findings suggest that lactate or lactylation might influence the hydroxylation of HIF-1α. However, lactate or sodium lactate treatment did not alter the hydroxylated HIF-1α level. In addition, the lactylation of HIF-1α does not influence its hydroxylation, and hydroxylated HIF-1α can undergo lactylation. However, increased lactylation at the K644/K12 site of hydroxylated HIF-1α inhibits its binding to VHL. In contrast, changes in the hydroxylation modification at proline sites of lactylated HIF-1α do not affect its binding to VHL. These findings suggest that lactylation modification of HIF-1α could inhibit its binding to VHL, irrespective of whether hydroxylation modification occurs. Additional investigations have shown that lactylation of HIF-1α impedes its degradation by obstructing the interaction between hydroxylated HIF-1α and VHL. Based on these findings, we propose the groundbreaking concept that HIF-1α lactylation might exert an “epistatic effect” on the classical PHD–hydroxylation pathway.

In this study, we identified two non-ODD lactylation sites—K644 in mouse and K12 in human and porcine HIF-1α—whose modification disrupts VHL binding and enhances HIF-1α stability. Although these sites lie outside the canonical VHL-interacting region, distal modifications frequently modulate HIF-1α turnover. For example, SUMOylation at K391—just upstream of the ODD—stabilizes HIF-1α [27], and its phosphorylation at S641/S643 enhances its nuclear retention [28]. Likewise, in other transcription factors, modifications far from core interaction motifs exert long-range allosteric effects. Likewise, long-range effects of distal modifications have been documented in other transcription factors—p53 Ser15 phosphorylation and Lys382 acetylation enhance DNA binding despite their distance from the core domain [29–31], FOXO3a Ser253 phosphorylation regulates 14-3-3 binding and localization [32], and STAT3 Lys685 acetylation stabilizes dimerization outside its SH2 domain [33]. To test whether lactylation exerts a similar allosteric influence, we used AlphaFold3 to model HIF-1α in both its unmodified form and with a lactyl moiety at K12 (human) or K644 (mouse). These models reveal that lactylation induces subtle conformational shifts that propagate toward the ODD, altering its topology and reducing the spatial accessibility of key hydroxylation sites (P402/P564 in humans; P577 in mice) (Fig. S11F-G, Fig. S13C). This remodeling provides a plausible mechanism by which lactylation at a distal lysine impairs VHL recognition and enhances HIF-1α stability. Future biophysical studies—such as hydrogen–deuterium exchange mass spectrometry—will be necessary to confirm this long-range regulatory model.

Hypoxia promotes the nuclear accumulation of HIF-1α and increases its lysine lactylation. This finding suggests that lactylation may affect the subcellular localization of HIF-1α. HIF-1α trafficking is governed by two nuclear localization signals (NLSs) [18] and a phosphorylation-sensitive nuclear export signal (NES, aa 632–639) embedded within the MAPK target domain (aa 616–658) [28, 34]. Phosphorylation of Ser641/Ser643 by ERK1/2 inhibits NES activity, whereas mutations in hydrophobic NES residues also block export. Although K644 does not fall within either NLS, it is adjacent to the NES and lies within the MAPK target domain. Given this proximity, it is plausible that modifications at K644 could affect NES function. However, our experimental results clearly show that K644 lactylation does not influence HIF-1α localization, indicating that it likely does not interfere with the activity of NLS, NES, or MAPK-regulated phosphorylation events.

The transcriptional activity of HIF-1 is determined by not only the intrinsic functional domains of HIF-1α but also the chromatin environment at its target loci. HIF-1α contains two key transactivation domains—N-TAD (aa 482–601) and C-TAD (aa 786–826)—which recruit coactivators such as p300, CBP, and SRC-1 to drive gene expression [35–37]. The C-TAD is negatively regulated by FIH-1–mediated hydroxylation of Asn803, which blocks coactivator binding [38]. Although K644 is located outside these canonical domains and does not overlap with the DNA-binding or ARNT-dimerization regions, our results demonstrate that lactylation at this site markedly enhances the transcriptional activity of HIF-1α. This finding points to a noncanonical mechanism of regulation. One possibility is that K644 lactylation facilitates the recruitment of additional transcriptional coregulators or chromatin-modifying enzymes. HIF-1α is known to interact with both activators and repressors of transcription, such as BRG1, SIRT6, and EZH2 [39, 40], depending on the chromatin context. Lactylation at K644 may alter these interactions or promote the formation of new transcriptional complexes, even in the absence of direct involvement in classical activation domains. Further studies are needed to define how K644 lactylation influences coregulator recruitment, chromatin accessibility, or promoter specificity at HIF target genes.

In light of the emerging role of lactate as a signaling metabolite, which not only inhibits HDACs to increase H3/H4 acetylation and DNA repair [41] but also directly lactylates transcription factors such as p53 to alter their activity [42], we propose that HIF-1α lactylation similarly reprograms key hypoxia-driven phenotypes. In tumor-like, lactate-rich hypoxic environments, the stabilization of lactylated HIF-1α may potentiate glycolytic and prosurvival gene programs (e.g., VEGF and GLUT1), thereby enhancing metabolic adaptation, cell survival, and chemoresistance [43, 44]. It can also amplify the expression of EMT markers and matrix metalloproteinases to drive invasion and migration. This insight may help further elucidate the molecular basis of HIF-1α-mediated tumor progression and may offer new therapeutic targets for hypoxia-related malignancies. In addition to its role in cancer, HIF-1α also plays essential roles in physiological hypoxic responses. Our previous work demonstrated that HIF-1α is indispensable for angiogenesis during follicular development and that its inhibition impairs follicular vascularization and ovulation [45]. Based on the current findings, enhancing HIF-1α stability through lactylation may represent a novel strategy for promoting follicle maturation and ovulation. Importantly, HIF-1α can exert both prosurvival and proapoptotic effects depending on the cellular context. For example, it promotes cell survival under hypoxia by upregulating antiapoptotic genes such as BCL2 [46, 47] and by inducing protective autophagy via BNIP3 [48]. Conversely, it can also initiate apoptosis via the activation of proapoptotic genes such as p53 [49]. Given the diverse and context-dependent roles of HIF-1α, fully resolving the phenotypic impact of its lactylation under hypoxia is technically beyond the scope of this study. However, our mechanistic dissection of how lactylation stabilizes HIF-1α provides a clear conceptual framework for future investigations into the potential roles of lactylated HIF-1α in mediating hypoxia- or lactate-associated cellular phenotypes.

**Fig. 8:**
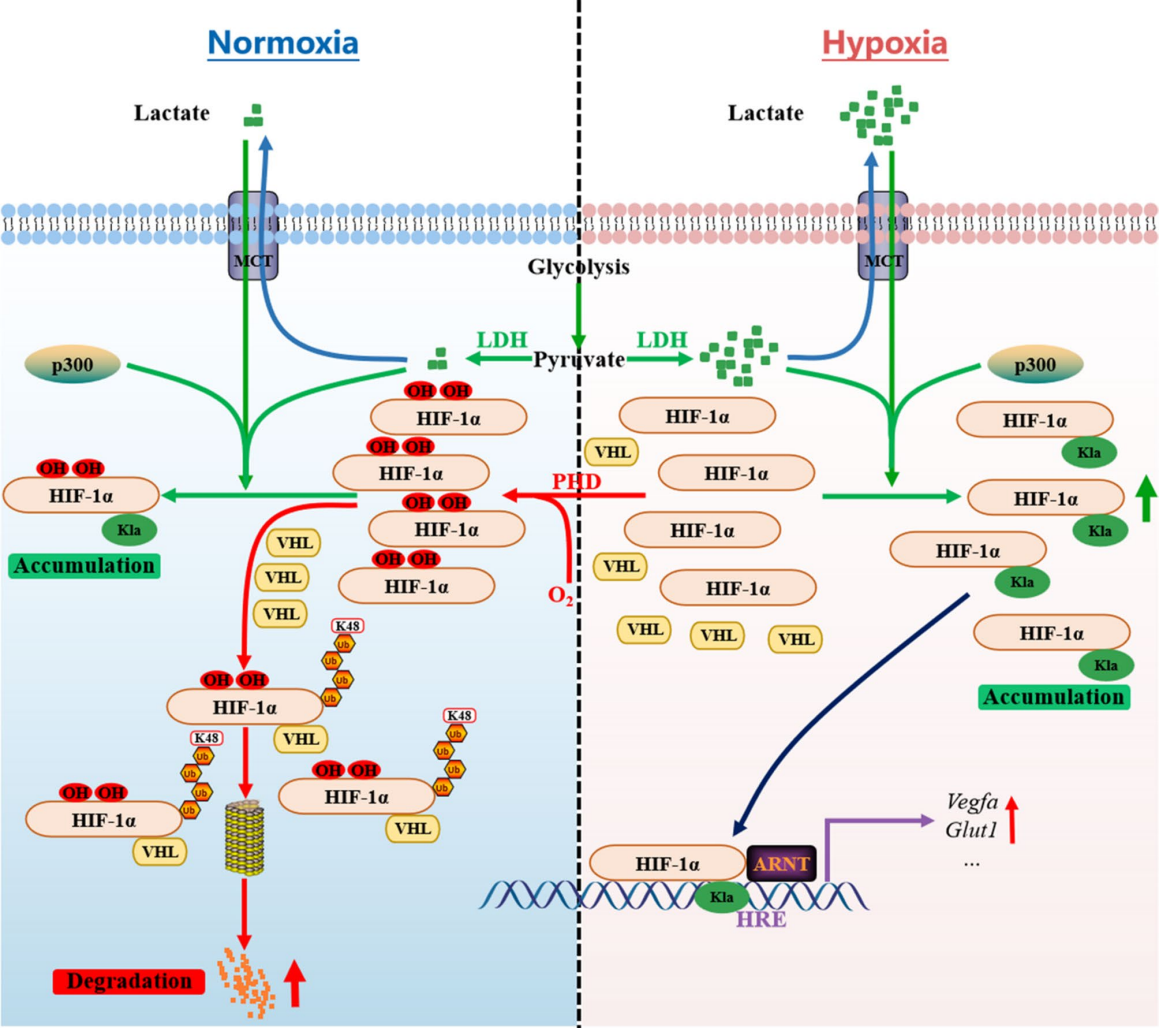
A proposed model illustrating how lactylation and hydroxylation collaborate to regulate HIF-1α protein stability under normoxic/hypoxic conditions. Under normoxic conditions, HIF-1α is modified via hydroxylation and catalyzed by prolyl hydroxylases (PHDs), enabling its recognition by the E3 ubiquitin ligase VHL and subsequent K48-linked ubiquitination, followed by degradation via the proteasomal pathway. However, cellular metabolism and MCT uptake generate small amounts of lactate intracellularly. Catalyzed by p300, lactate promotes the lactylation of hydroxylated HIF-1α, causing its dissociation from VHL. Consequently, HIF-1α evades ubiquitination and undergoes limited accumulation. Under hypoxic conditions, diminished oxygen levels result in reduced proline hydroxylation and HIF-1α protein stabilization. Concurrently, anaerobic glycolysis facilitates lactate production, leading to extensive lactylation of HIF-1α, causing substantial accumulation of the HIF-1α protein and thus promoting the transcription of downstream target genes

In summary, our data support a working model in which HIF-1α K644la/K12la improves HIF-1α protein stability via two distinct mechanisms: (1) by directly inhibiting VHL recognition of HIF-1α and (2) by shielding VHL from recognizing hydroxylated HIF-1α. Under normoxic conditions, increased binding between VHL and HIF-1α occurs for two reasons: (1) the low level of HIF-1α lactylation under normoxic conditions results in minimal shielding of hydroxylation, allowing it to promote VHL binding; and (2) the low level of HIF-1α lactylation alleviates its inhibition of VHL recognition, promoting VHL binding. Conversely, under hypoxic conditions, the decreased binding between VHL and HIF-1α is attributed to the following: (1) the high level of HIF-1α lactylation during hypoxic conditions shields it from hydroxylation, preventing it from exerting its effect on VHL binding; and (2) HIF-1α lactylation itself blocks the VHL recognition process, thus suppressing VHL binding (Fig. 8).

## Supplementary Information

Below is the link to the electronic supplementary material.

Supplementary Material 1

Supplementary Material 2

## Data Availability

No datasets were generated or analysed during the current study.
