# Supplementary material for: Lactylation modification of HIF-1α enhances its stability by blocking VHL recognition
Source: Cell Commun Signal. 2025 Aug 4;23:364. doi: 10.1186/s12964-025-02366-x (PMC12323271; doi:10.1186/s12964-025-02366-x)
Supplement: Supplementary file 1 — Supplementary Material 1 [file 12964_2025_2366_MOESM1_ESM.doc]

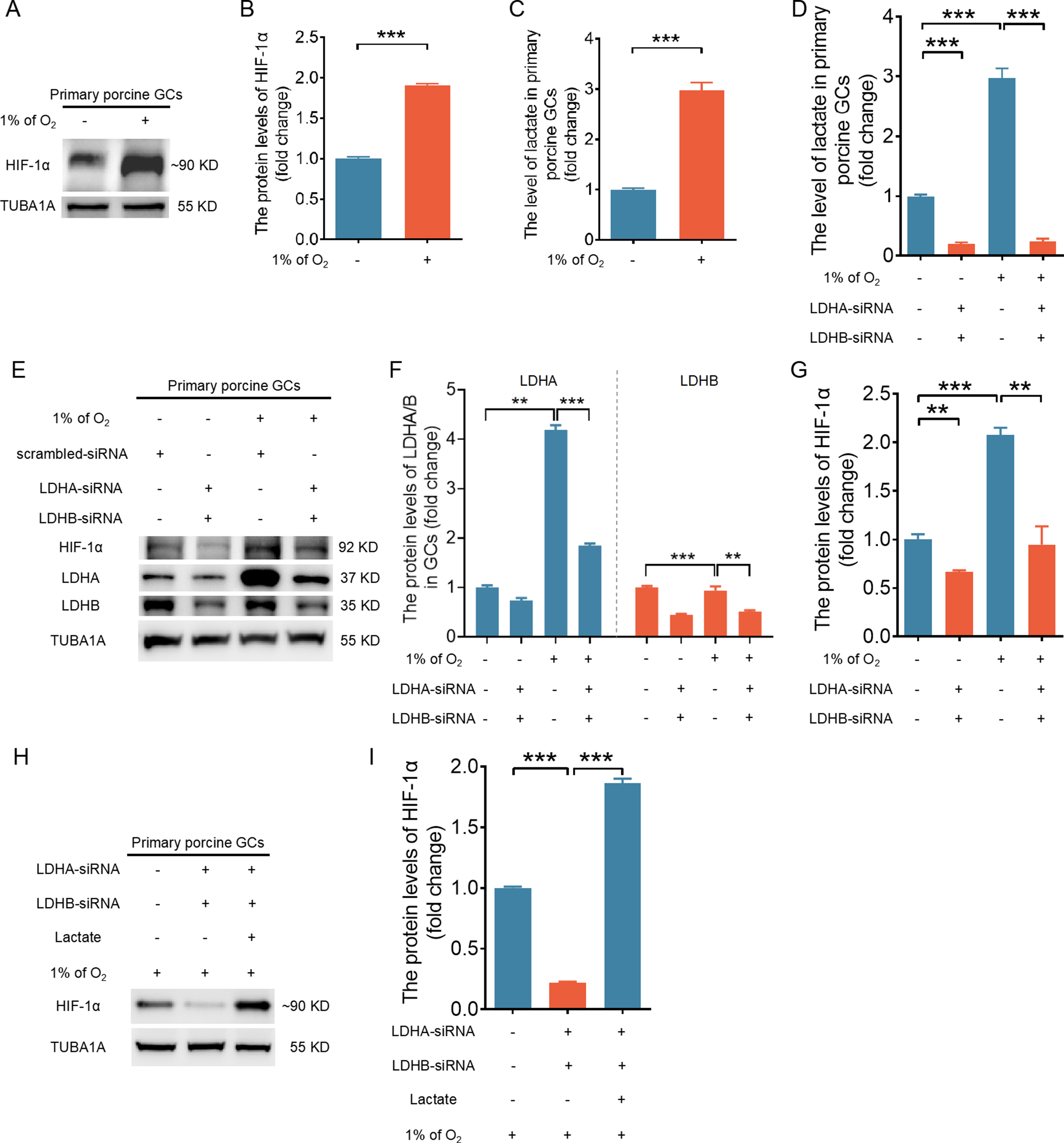


**Figure S1. Lactate contributes to HIF-1α protein accumulation in hypoxic primary porcine granulosa cells (GCs)**. A–C, Primary porcine GCs were cultured under normoxic (21% O_2_) or hypoxic (1% O_2_) conditions for 6 h. The cells were then harvested to assess HIF-1α protein levels (A and B) and intracellular lactate concentrations (C). D–G, GCs were transfected with siRNAs targeting LDHA and LDHB or a scramble control for 24 h, followed by 6 h of culture under normoxic or hypoxic conditions. Lactate levels (D) and protein levels of HIF-1α, LDHA, and LDHB (E) were determined. The quantification of the bands is shown in (F) and (G). H and I, GCs transfected with LDHA/LDHB siRNAs or scramble control siRNA were treated with or without 1 mM sodium lactate and cultured under hypoxic conditions for 6 h. HIF-1α protein levels were analyzed by western blotting (H) and quantified (I). The data are presented as the means ± s.e.m.s (n = 3). **P < 0.01, ***P < 0.001.


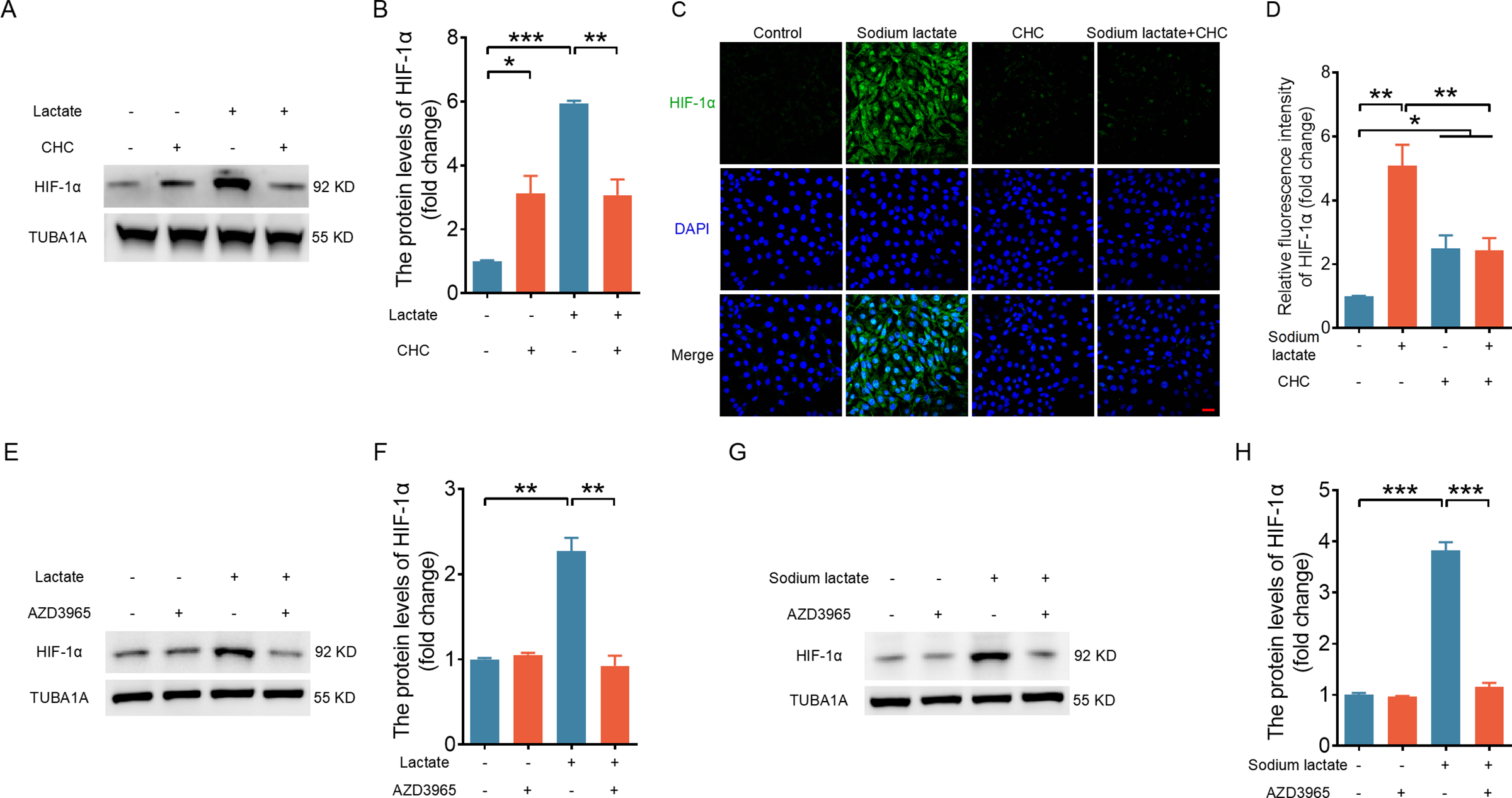


**Figure S2. MCT inhibitors CHC and AZD3965 block sodium lactate-induced accumulation of HIF-1α.** A–D, NIH/3T3 cells were pretreated with 3 mM CHC for 2 h, followed by incubation with 1 mM sodium lactate for an additional 6 h. The cells were then collected for western blot analysis of HIF-1α (A and B) or subjected to immunofluorescence staining to assess HIF-1α subcellular localization (C). The HIF-1α fluorescence intensity was quantified using ImageJ (D). Scale bar, 50 μm. E–H, NIH/3T3 cells were pretreated with or without 80 μM AZD3965 for 2 h and then incubated with 10 mM lactate (E and F) or 1 mM sodium lactate (G and H) for an additional 6 h. The cells were harvested for western blot analysis of HIF-1α (E and G), and the band intensities were quantified (F and H). The data are presented as the means ± s.e.m.s (n = 3). **P* < 0.05, ***P* < 0.01, ****P* < 0.001.


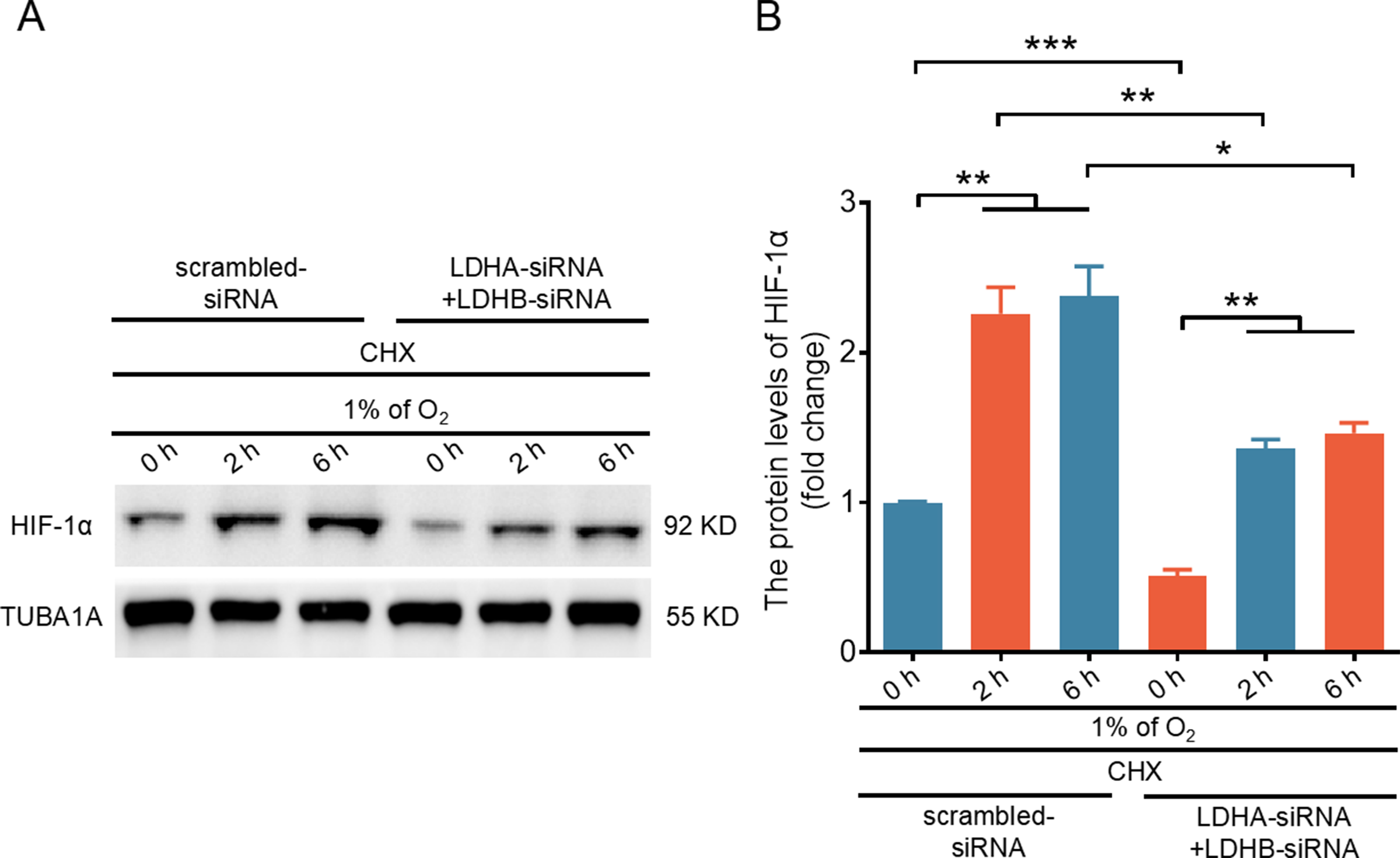


**Figure S3. LDHA and LDHB knockdown attenuates hypoxia-induced HIF-1α accumulation under translational inhibition.** A and B, NIH/3T3 cells were transfected with LDHA/LDHB siRNAs or scramble control siRNA for 24 h, followed by culture under hypoxic conditions (1% O₂) for an additional 0, 2, or 6 h. Cells were then collected for western blot analysis of HIF-1α protein levels (A), and the bands were quantified (B). The data are presented as the means ± s.e.m.s (n = 3). **P* < 0.05, ***P* < 0.01, ****P* < 0.001.


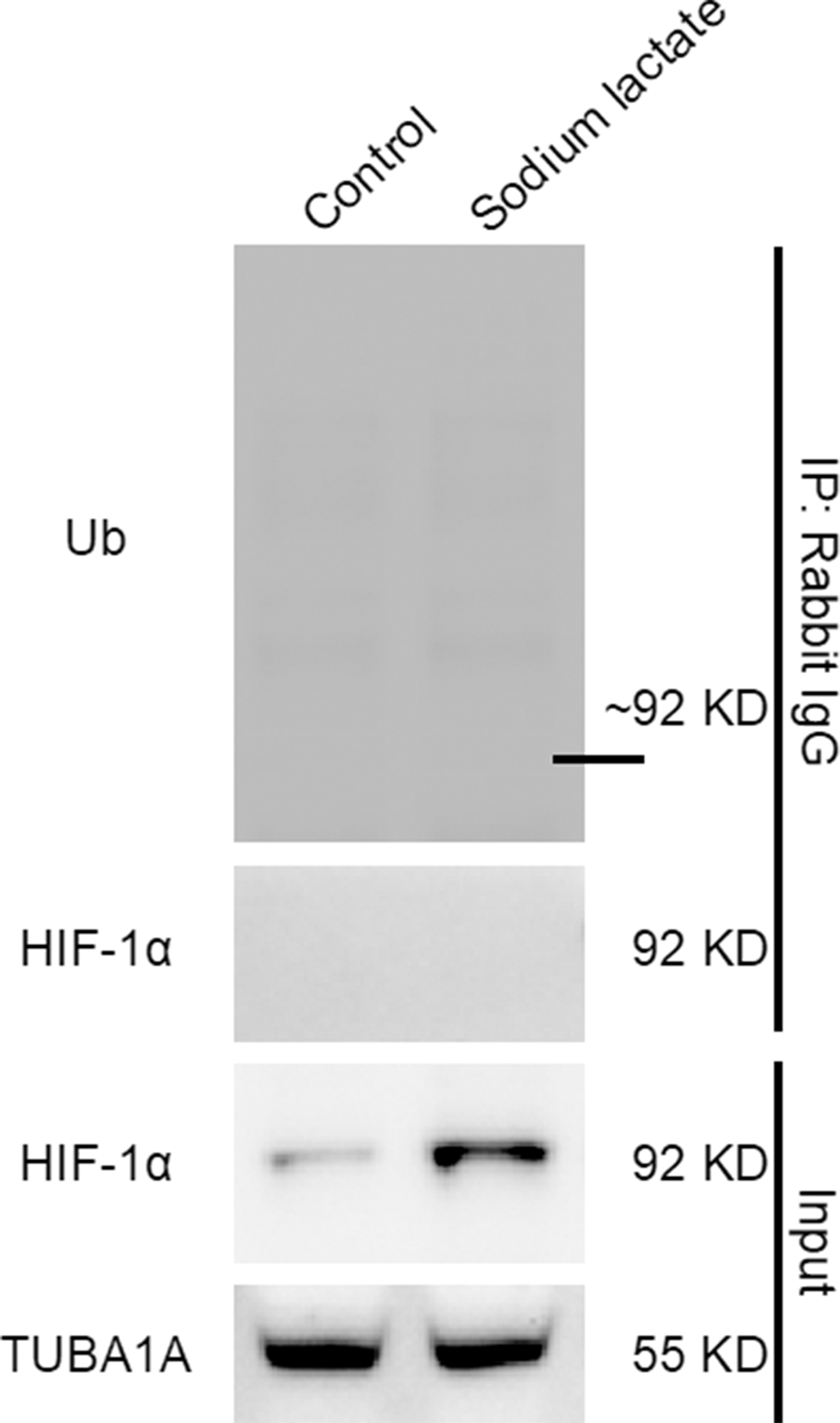


**Figure S4. Sodium lactate reduces HIF-1α ubiquitination under normoxia.** NIH/3T3 cells were cultured under normoxic conditions (21% O_2_) and treated with 1 mM sodium lactate for 6 hours. Immunoprecipitation (IP) was then performed to assess the levels of HIF-1α and ubiquitin, with rabbit IgG used as a negative control.


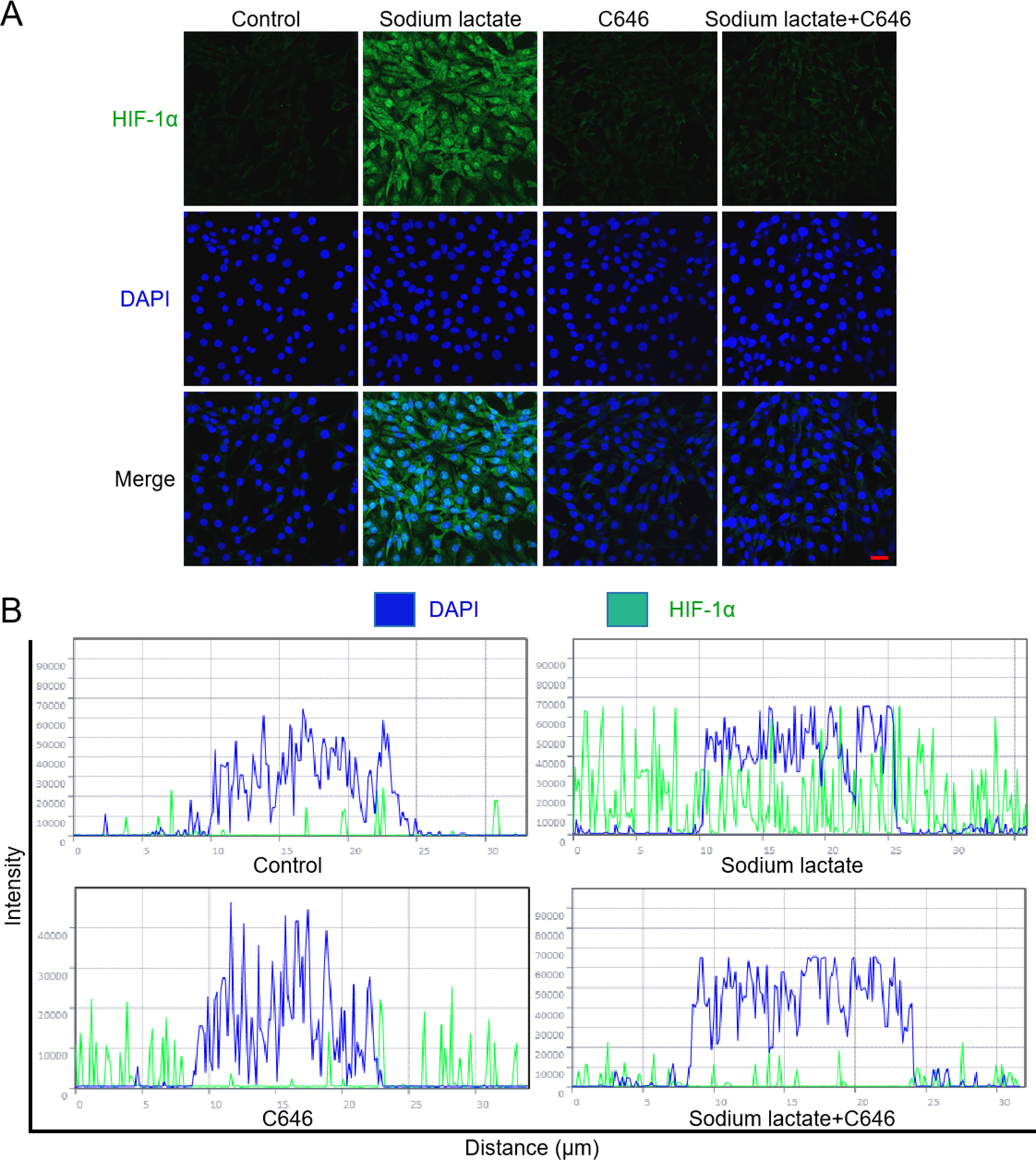


**Figure S5. The P300 inhibitor C646 abrogates the sodium lactate-induced accumulation of the HIF-1α protein.** A, NIH/3T3 cells pretreated with 10 μM C646 for 2 h were cultured with 1 mM sodium lactate for 6 h and then collected to observe the subcellular localization of HIF-1α via immunofluorescence. The scale bar represents 50 μm. B, The fluorescence intensity curves show the distributions of HIF-1α (green) and DAPI (blue) in the cells.


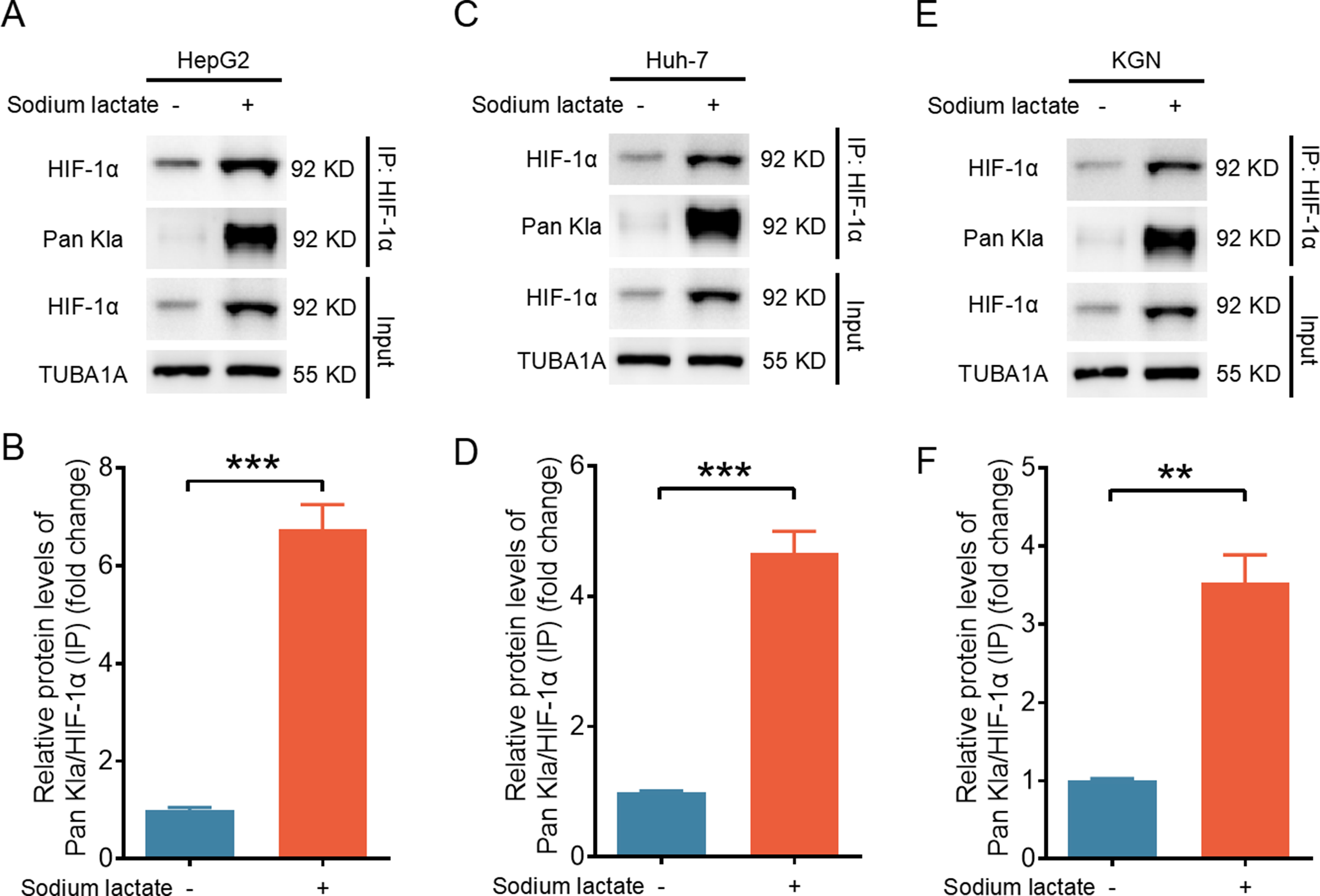


**Figure S6. Sodium lactate promotes HIF-1α lactylation in HepG2, Huh-7, and KGN cells.** HepG2 (A, B), Huh-7 (C, D), or KGN (E, F) cells were treated with 1 mM sodium lactate for 6 h. HIF-1α lactylation levels were assessed by immunoprecipitation followed by western blotting. The data are presented as the means ± s.e.m.s (n = 3). ***P* < 0.01, ****P* < 0.001.


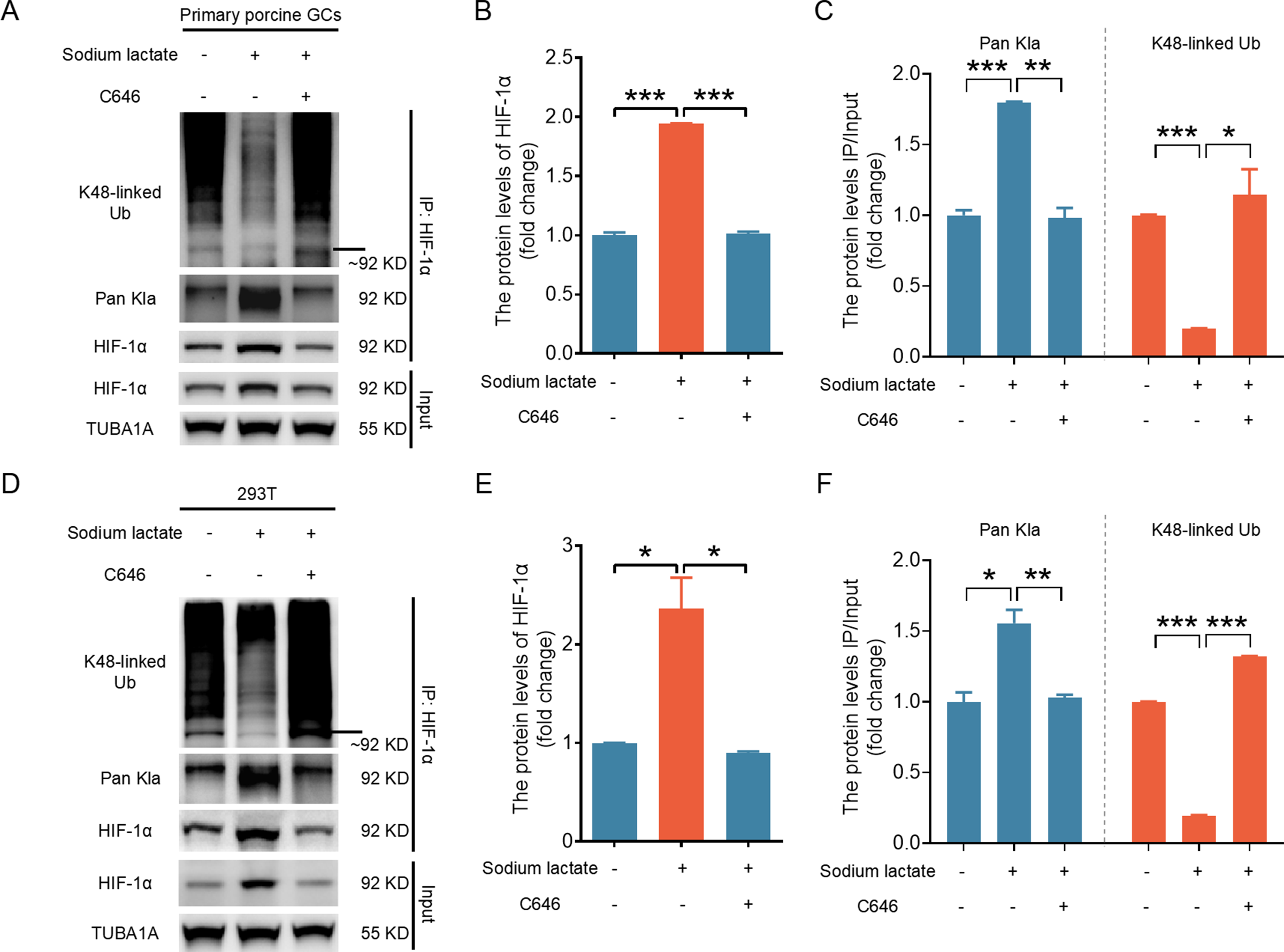


**Figure S7. The suppression of HIF-1α lactylation by C646 treatment restores HIF-1α ubiquitination in porcine GCs and 293T cells.** A–F, Primary porcine GCs (A–C) and 293T cells (D-F) pretreated with 10 μM C646 for 2 h were cultured with 1 mM sodium lactate for 6 h. The lactylation and K48-linked ubiquitination levels of the HIF-1α protein were determined by IP and quantified. The data are presented as the means ± s.e.m.s (n = 3). **P* < 0.05, ***P* < 0.01, ****P* < 0.001.


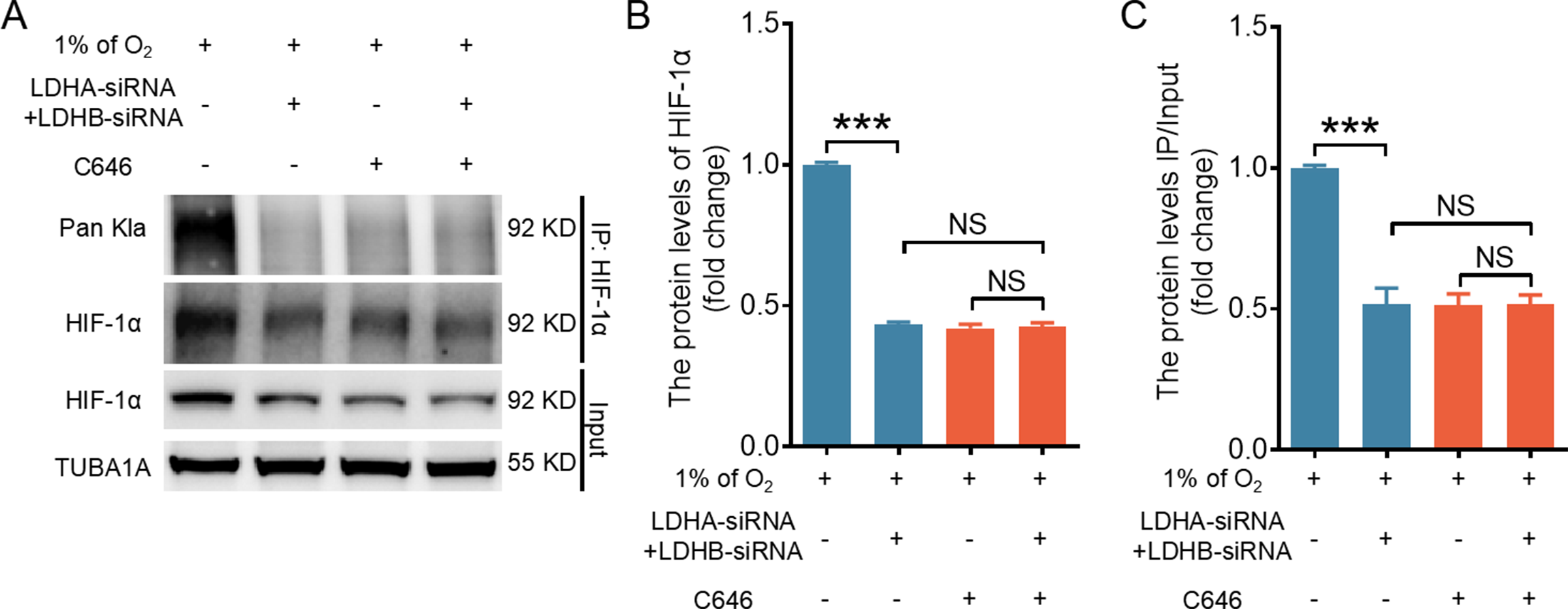


**Figure S8. Knockdown of LDHA and LDHB prevents further inhibition of HIF-1α accumulation and lactylation by C646.** A–C, NIH/3T3 cells pretreated with 10 μM C646 were transfected with LDHA/LDHB siRNAs for 24 h and then cultured under hypoxia (1% O_2_) for 6 h. IP was performed to determine the lactylation level of the HIF-1α protein (A), and the results were quantified (B and C). The data are presented as the means ± s.e.m.s (n = 3). ****P* < 0.001; NS, not significant (*P* > 0.05).


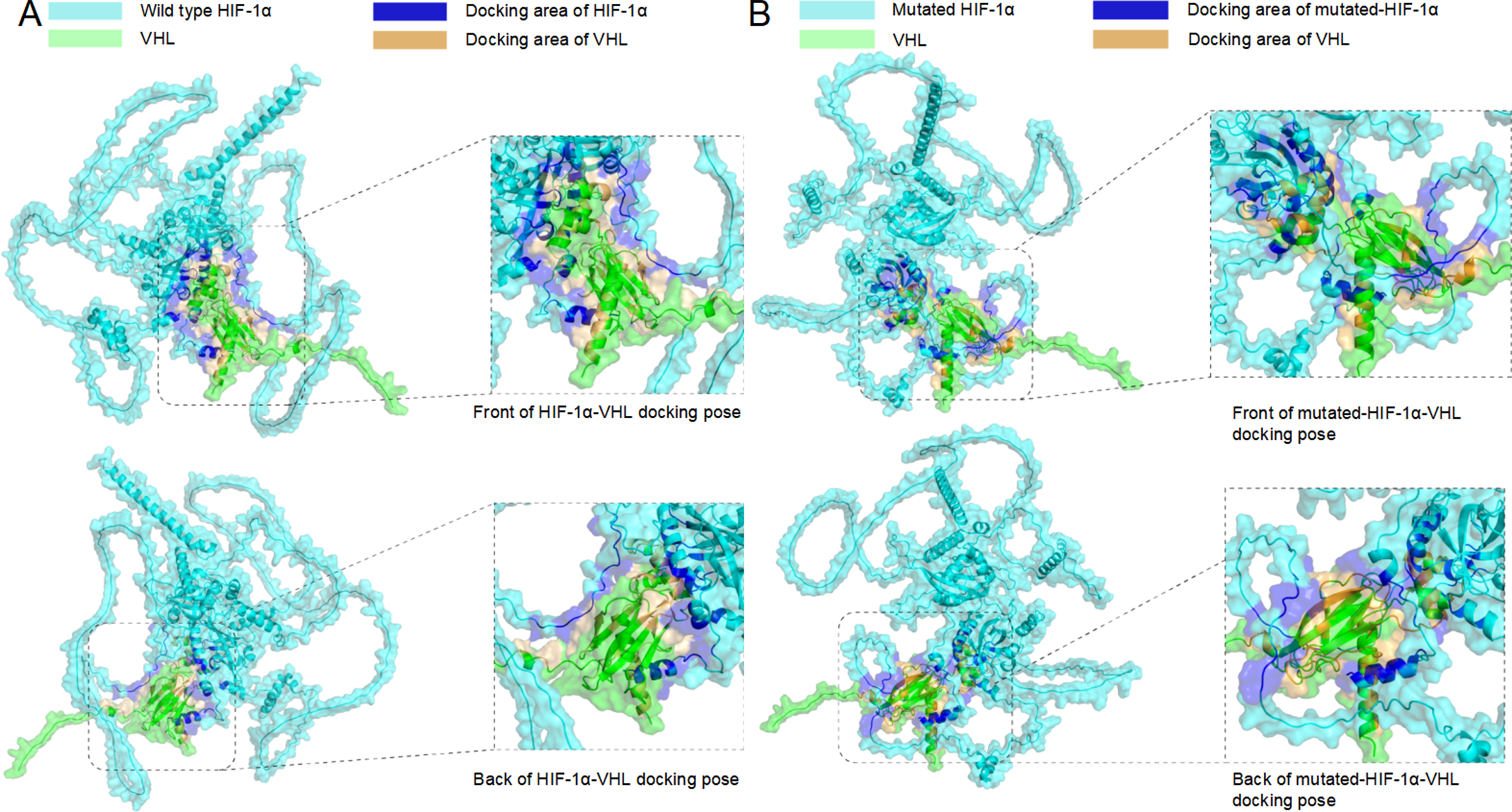


**Figure S9. Molecular docking analysis of HIF-1α and VHL interactions.** A and B, Three-dimensional structural models of the wild-type (WT) HIF-1α, K644R mutant HIF-1α, and VHL proteins were generated using AlphaFold3 based on structural information from UniProt (HIF-1α: Q61221; VHL: P40338). Molecular docking of WT or K644R mutant HIF-1α with VHL was performed using ClusPro 2.0. The docking model with the highest binding affinity (lowest energy score) was selected, and the results were visualized using PyMOL.


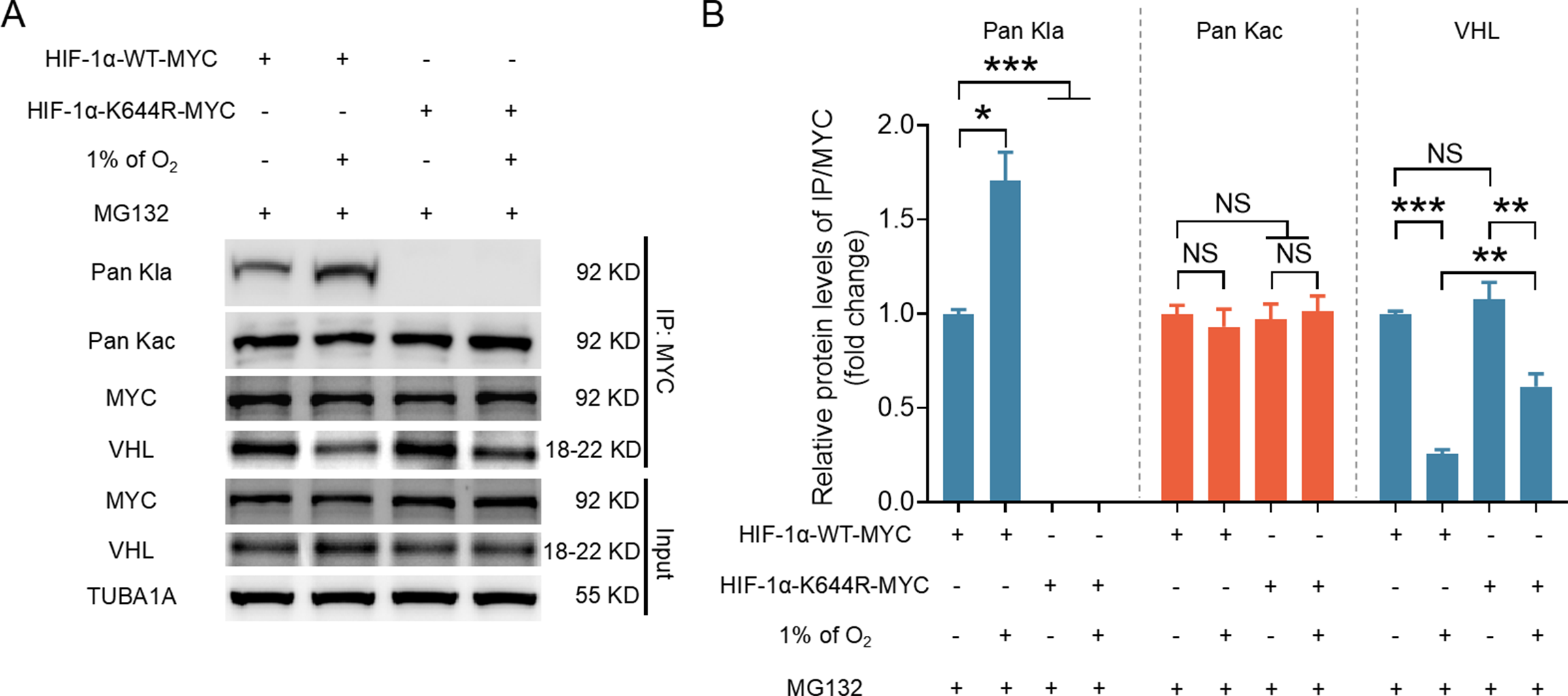


**Figure S****10. The K644R mutation restores the interaction between HIF-1α and VHL under hypoxia by preventing lactylation.** A and B, NIH/3T3 cells were transfected with MYC-tagged wild-type HIF-1α or the K644R mutant for 24 h. The cells were then pretreated with 10 μM MG132 for 2 h and exposed to hypoxia (1% O2) for 6 h. Western blotting was performed to detect the MYC and VHL protein levels (A). IP was used to assess the interaction between MYC and VHL, as well as the lactylation and acetylation levels of MYC-tagged HIF-1α (A). The quantified data are shown in (B). The data are presented as the means ± s.e.m.s (n = 3). **P* < 0.05, ***P* < 0.01, ****P* < 0.001; NS, not significant (*P* > 0.05).


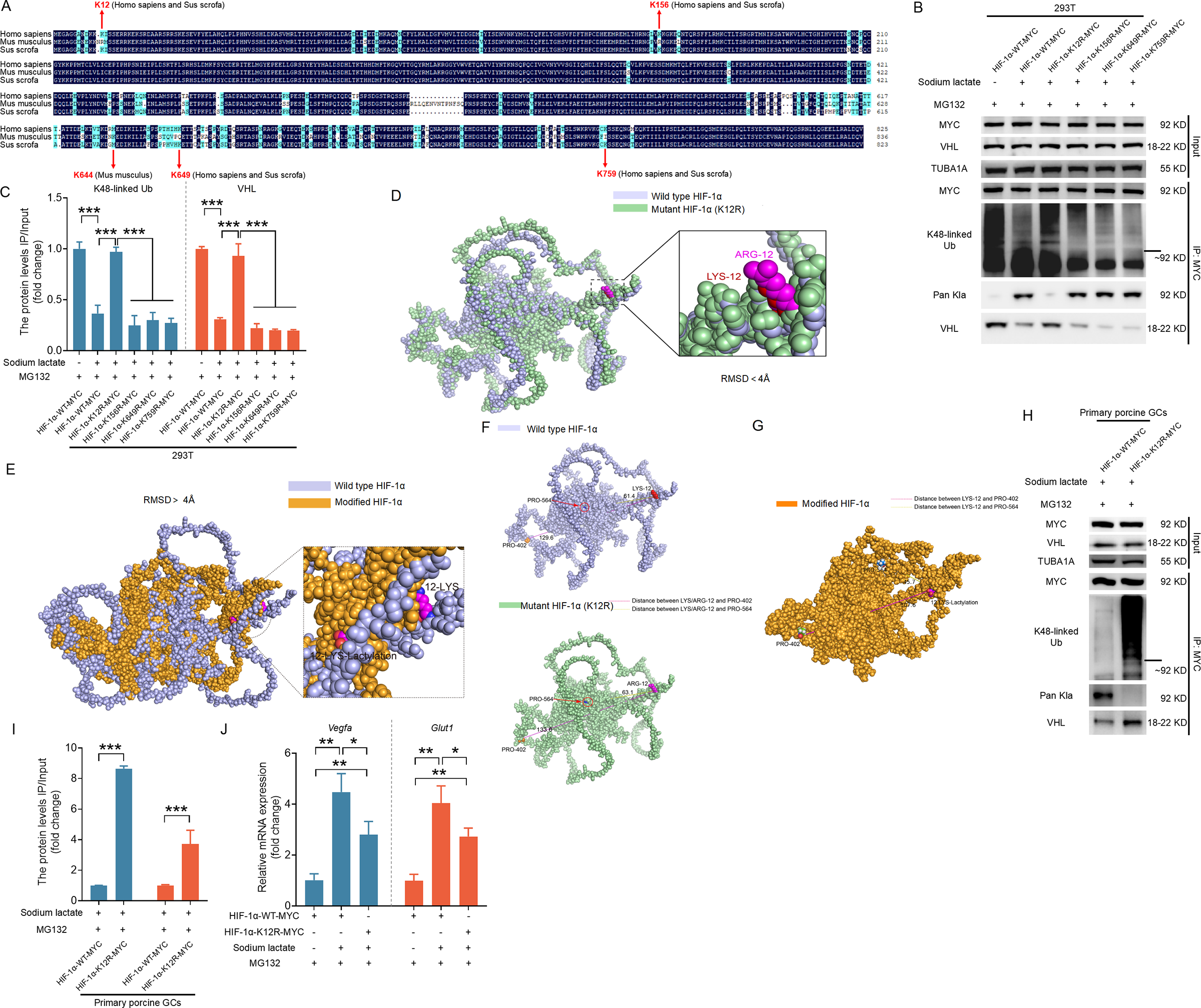


**Figure S11. K12 serves as a conserved lactylation site in human and porcine HIF-1α, inhibiting K48-linked ubiquitination and VHL binding.** A, Amino acid sequences of human, porcine, and murine HIF-1α proteins were compared using DNAMAN software to assess sequence identity. The arrows indicate lysine residues that are not conserved between humans or pigs and mice. B and C, 293T cells were transfected with vectors encoding MYC-tagged wild-type HIF-1α or its K12R, K156R, K649R, or K759R mutants for 24 hours. After transfection, the cells were pretreated with 10 μM MG132 for 2 hours, followed by incubation with 1 mM sodium lactate for 6 hours. The levels of VHL binding to MYC-HIF-1α, as well as the lactylation and K48-linked ubiquitination of MYC-HIF-1α, were assessed by immunoprecipitation (IP) (B) and quantified (C). D and E, Structural comparison of wild-type HIF-1α with the K12R mutant (D) or the lactylated K12 variant (E), as predicted by AlphaFold3. The stick representations highlight the K12, lactylated K12, and R12 residues. Root mean square deviation (RMSD) values were calculated to assess structural alterations, with lower RMSD values indicating minimal conformational changes. F and G. Spatial distances between K12 (or R12, or lactylated K12) and P402, as well as between K12 (or R12, or lactylated K12) and P564, were analyzed within the three-dimensional structure of HIF-1α. Distances were measured using Cα atoms and visualized in PyMOL, with key residues highlighted to illustrate spatial relationships relative to known hydroxylation sites. H and I, Porcine granulosa cells (GCs) were transfected with vectors encoding MYC-tagged wild-type or K12R mutant HIF-1α for 24 hours. After transfection, the cells were pretreated with 10 μM MG132 for 2 hours, followed by incubation with 1 mM sodium lactate for 6 hours. The levels of VHL binding to MYC-HIF-1α, as well as the lactylation and K48-linked ubiquitination of MYC-HIF-1α, were determined by IP (H) and quantified (I). J, 293T cells pretreated with 10 μM MG132 for 2 h were transfected with HIF-1α-WT-MYC or HIF-1α-K12R-MYC for 24 h and then cultured with 1 mM sodium lactate for 6 h. The mRNA levels of *Vegfa* and *Glut1* were measured by qRT‒PCR. The data are presented as the means ± s.e.m.s (n = 3). **P* < 0.05, ***P* < 0.01, ****P* < 0.001.


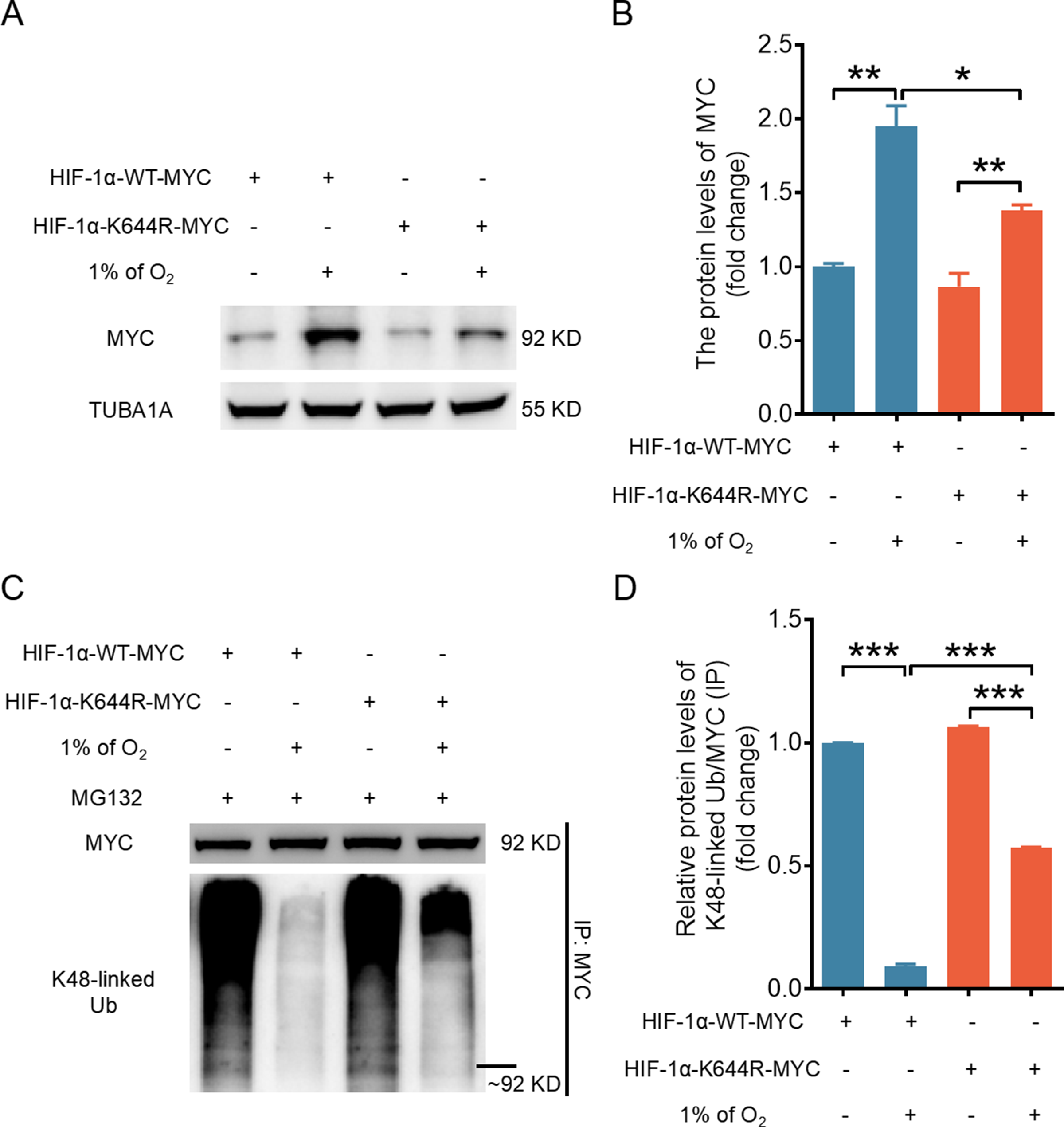


**Figure S12. The K644R mutation cannot completely inhibit HIF-1α accumulation and fails to fully restore HIF-1α ubiquitination under hypoxic conditions.** A–D, NIH/3T3 cells pretreated with or without 10 μM MG132 for 2 h were transfected with vectors encoding MYC-tagged wild-type HIF-1α or the K644R mutant for 24 h and then cultured under normoxic (21% O_2_) or hypoxic (1% O_2_) conditions for 6 h. The MYC protein level was measured by western blot (A) and quantified (B). IP was then performed to determine the K48-linked ubiquitination level of the MYC protein (C), and the results were quantified (D). The data are presented as the means ± s.e.m.s (n = 3). **P* < 0.05, ***P* < 0.01, ****P* < 0.001.


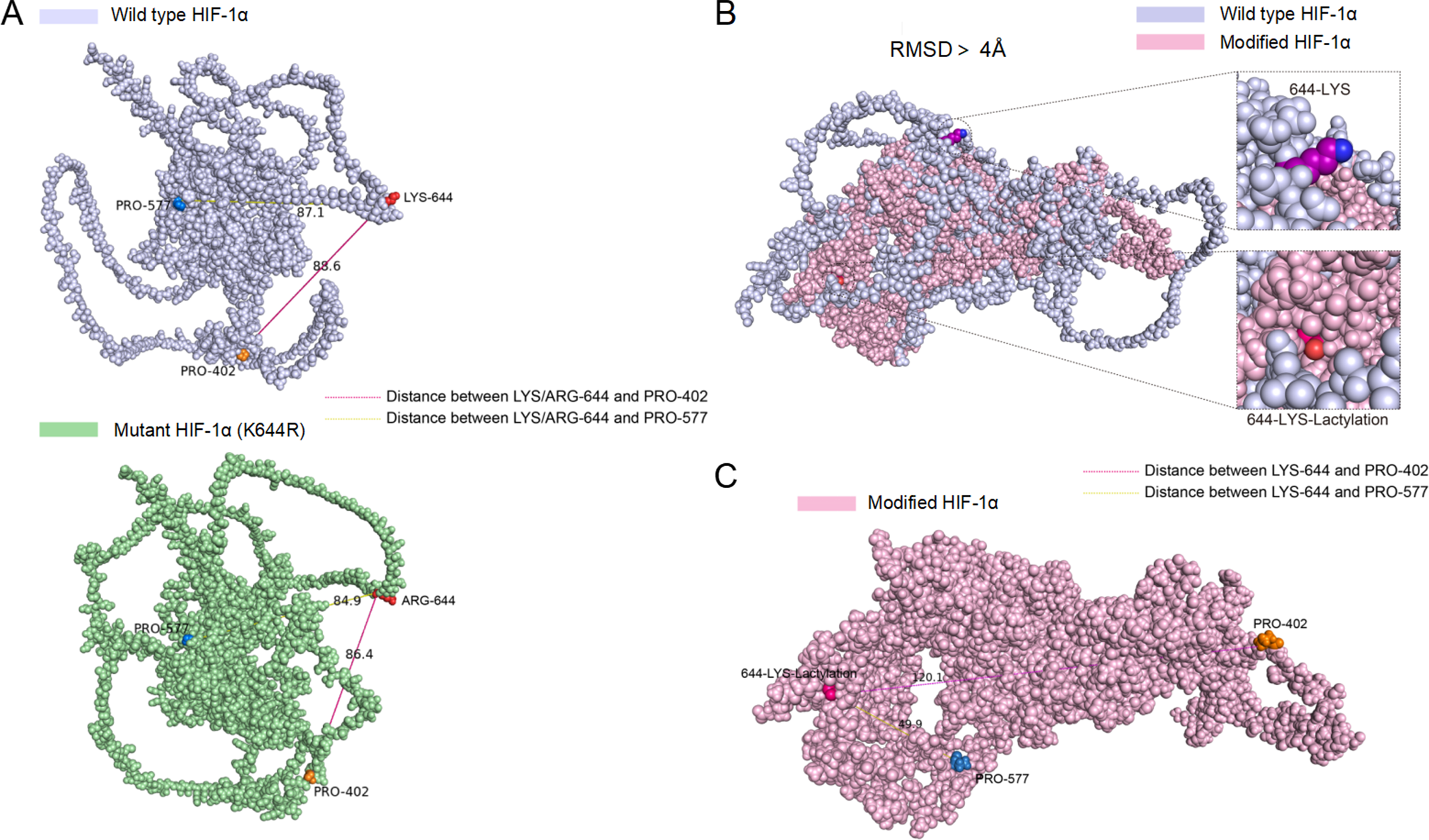


**Figure S13. Structural analysis of the spatial relationship between K644 and key hydroxylation sites within the HIF-1α protein.** A-C, The HIF-1α protein sequence (UniProt ID: P68040) was obtained from the UniProt database (https://www.uniprot.org/), and its three-dimensional structure was predicted using AlphaFold3. The distances between residue K644 (or R644 or lactylated K644) and P402 or P577 were measured and visualized using PyMOL, with key residues labeled for clarity. Measured distances between K644 (or R644) and P402 or P577 (A). Structural comparison of wild-type and K644-lactylated HIF-1α, with K644 and its lactylated form shown in stick representation. Root mean square deviation (RMSD) values were calculated to assess the structural impact, where lower RMSD values indicate less deviation from the native structure (B). Visualization of the distances between K644 (or lactylated K644) and P402 or P577 in the predicted three-dimensional structures (C).

**Supplementary Table S1** siRNA sequences.

| **siRNA Name** | **Sense（5'-3'）** | **Antisense（5'-3'）** |
| --- | --- | --- |
| *Scrambled siRNA*  *(SC)* | UUCUCCGAACGUGUCACGUTT | ACGUGACACGUUCGGAGAATT |
| *Ldha-siRNA*  Sus scrofa | GUGCUUAUGAGGUGAUCAATT | UUGAUCACCUCAUAAGCACTT |
| *Ldhb-siRNA*  Sus scrofa | GUGGAAGCUAAGUGGAUUATT | UAAUCCACUUAGCUUCCAGTT |
| *Ldha -siRNA*  Mus musculus | CACGUACACGGAGACCUCGGUAUUA | UAAUACCGAGGUCUCCGUGUACGUG |
| *Ldhb -siRNA*  Mus musculus | CCGUGUCUACCAUGGUGAAGGGAAU | AUUCCCUUCACCAUGGUAGACACGG |

**Supplementary Table S2** Primer sequences for RT-qPCR.

| **Gene Name** | **Primer Sequence (5'→3')** |
| --- | --- |
| *Tuba1a* | F: CCTAAACAGGTTGATAGGCCAAA  R: CTCGCCTTCCACAGAATCCA |
| *Hif-1α*  (mouse) | F: ACCTTCATCGGAAACTCCAAAG  R: ACTGTTAGGCTCAGGTGAACT |
| *Vegfa*  (mouse) | F: GCACATAGAGAGAATGAGCTTCC  R: CTCCGCTCTGAACAAGGCT |
| *Glut1*  (mouse) | F: CAGTTCGGCTATAACACTGGTG  R: GCCCCCGACAGAGAAGATG |
| *Vegfa*  (human) | F: CTGGAGCGTGTACGTTGGT  R: CAAGGCCCACAGGGATGGG |
| *Glut1*  (human) | F: TGGCATCAACGCTGTCTTCT  R: CTAGCGCGATGGTCATGAGT |

**Supplementary Table S3 Primer sequences for ChIP-qPCR.**

| **Gene Name** | **GenBank Accession NO.** | **Primer Sequence (5'→3')** |
| --- | --- | --- |
| *Vegfa*  Mus musculus | [GenBank: NC_000083.7] | F:CAAGCATAGAGAAATAGCCAAGGGT  R:TTGCCAGGGAACTGAAGCCAGGGTG |
