# Supplementary material for: Lactylation modification of HIF-1α enhances its stability by blocking VHL recognition
Source: Cell Commun Signal. 2025 Aug 4;23:364. doi: 10.1186/s12964-025-02366-x (PMC12323271; doi:10.1186/s12964-025-02366-x)
Supplement: Supplementary file 2 — Supplementary Material 2 [file 12964_2025_2366_MOESM2_ESM.pdf]

Fig. 1

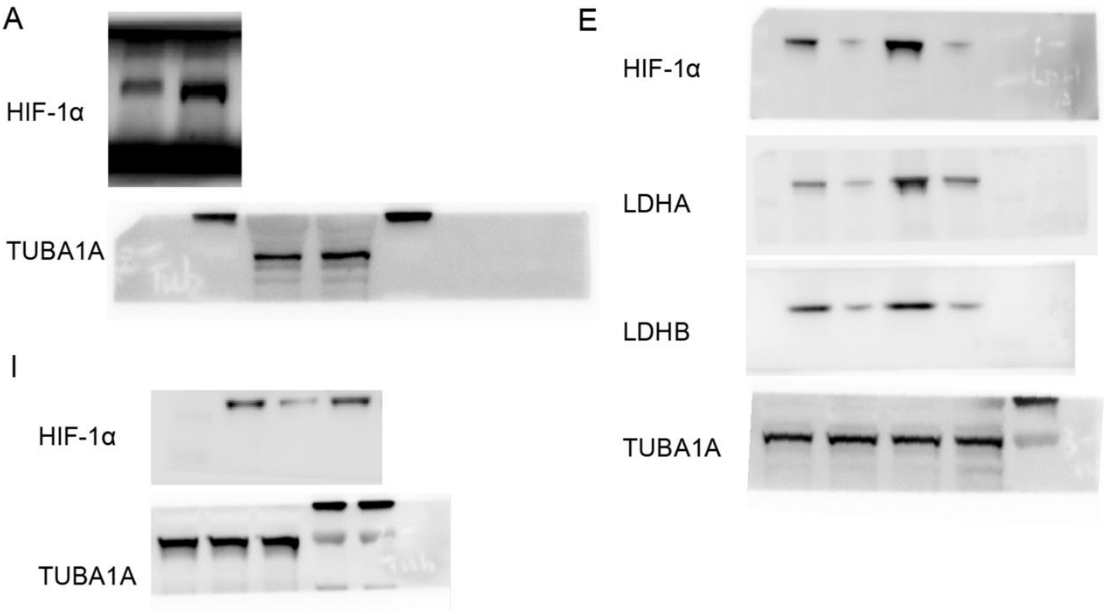

Fig. 2

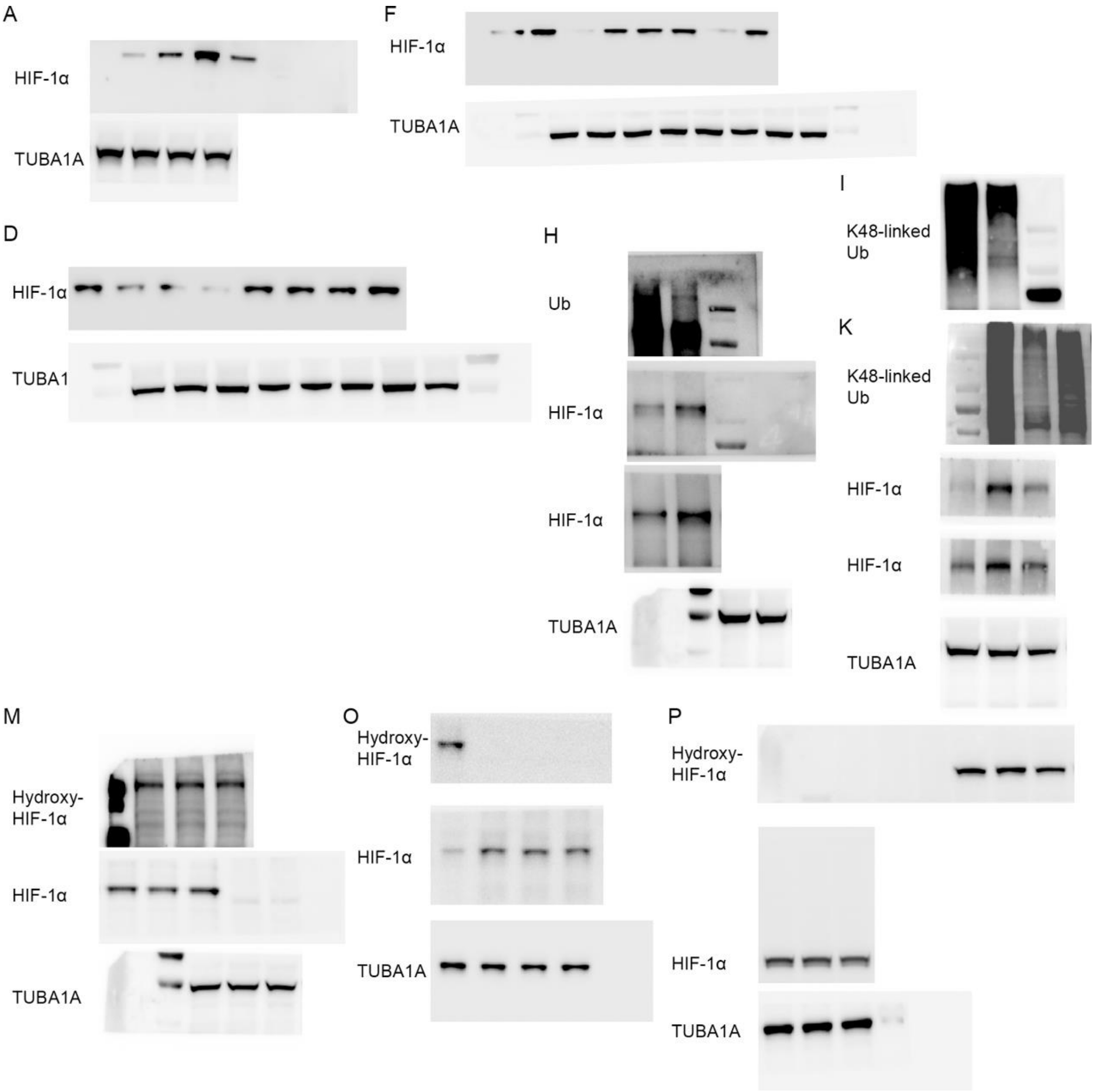
A
HIF-1α
TUBA1A
F
HIF-1α
TUBA1A
D
HIF-1α
TUBA1
H
Ub
HIF-1α
HIF-1α
TUBA1A
I
K48-linked Ub
K
K48-linked Ub
HIF-1α
HIF-1α
TUBA1A
M
Hydroxy-HIF-1α
HIF-1α
TUBA1A
O
Hydroxy-HIF-1α
HIF-1α
TUBA1A
P
Hydroxy-HIF-1α
HIF-1α
TUBA1A

Fig. 3

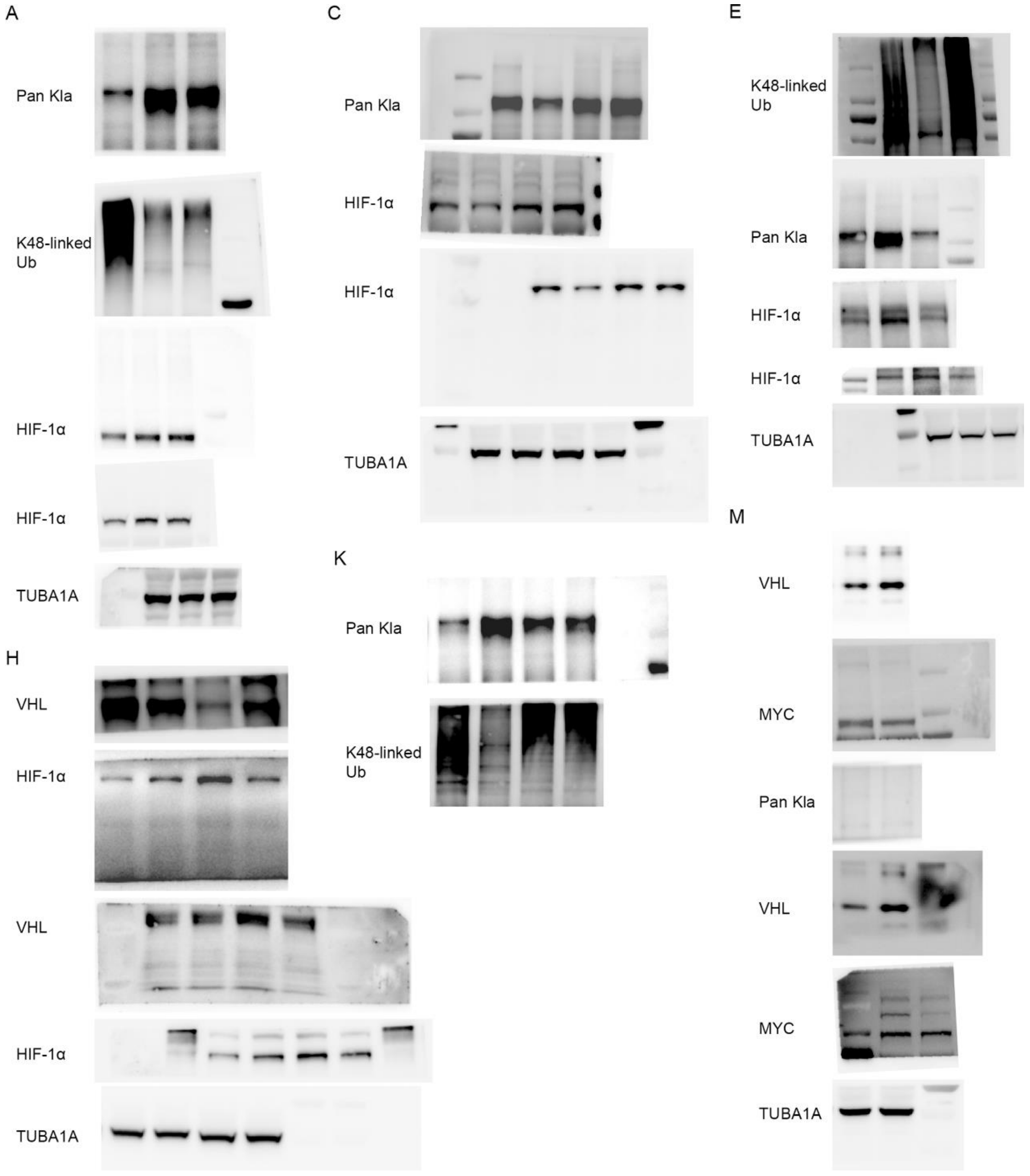
A
Pan Kla
K48-linked Ub
HIF-1α
HIF-1α
TUBA1A
C
Pan Kla
HIF-1α
HIF-1α
TUBA1A
E
K48-linked Ub
Pan Kla
HIF-1α
HIF-1α
TUBA1A
H
VHL
HIF-1α
VHL
HIF-1α
TUBA1A
K
Pan Kla
K48-linked Ub
M
VHL
MYC
Pan Kla
VHL
MYC
TUBA1A

Fig. 4

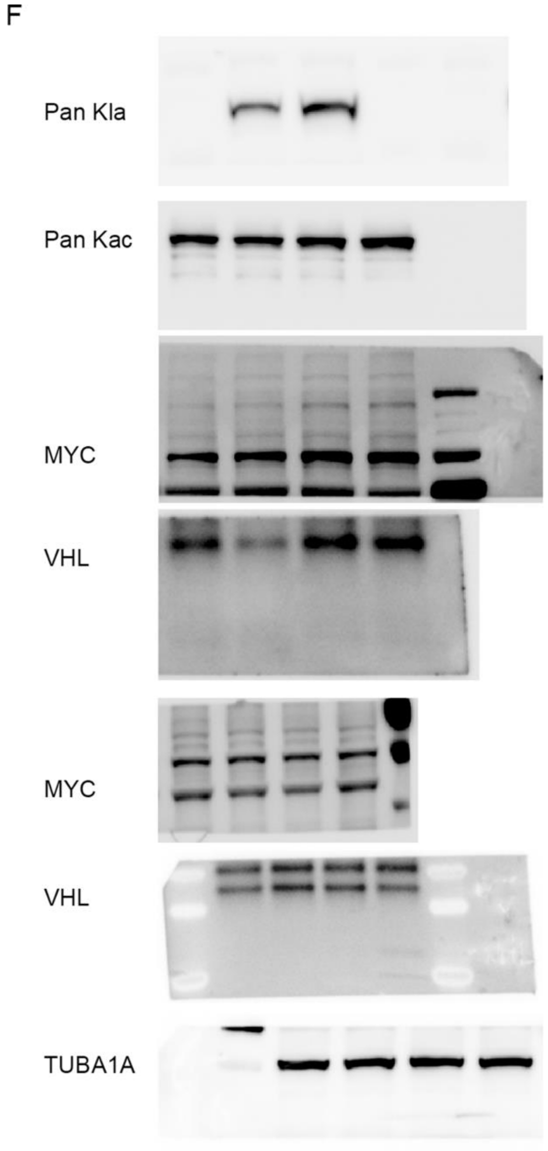

Fig. 5

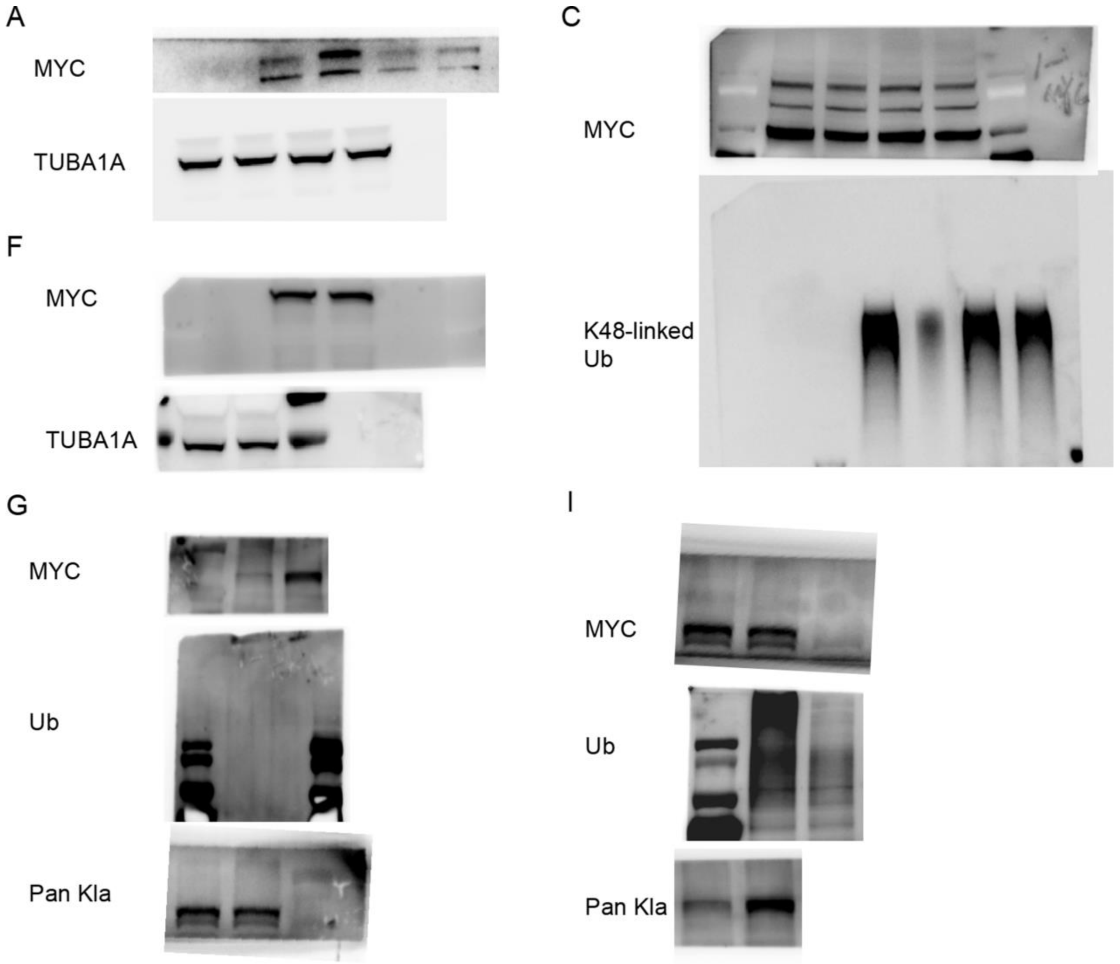
A
MYC
TUBA1A
C
MYC
K48-linked Ub
F
MYC
TUBA1A
G
MYC
Ub
Pan Kla
I
MYC
Ub
Pan Kla

Fig. 6-1

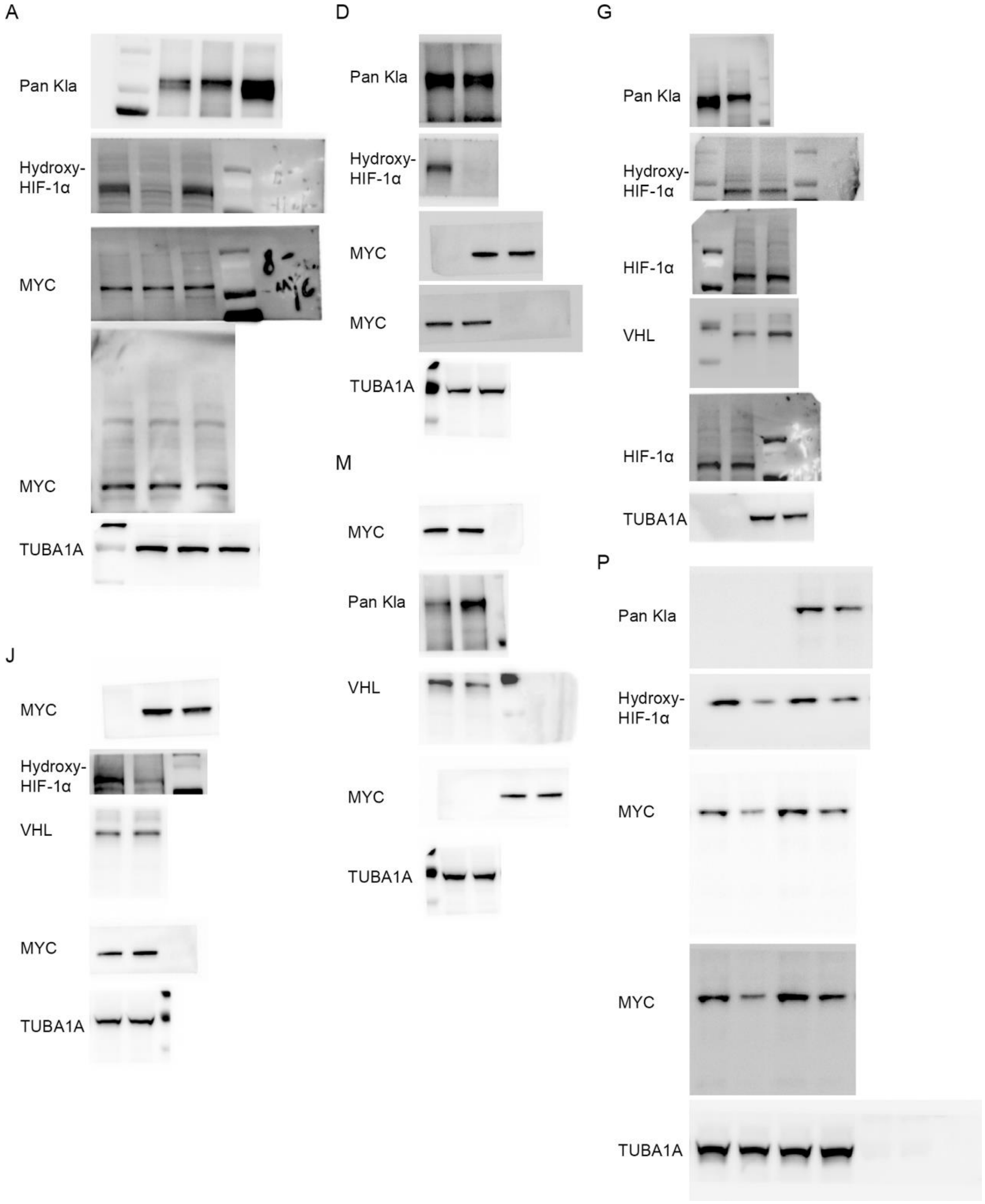
A
Pan Kla
Hydroxy-HIF-1α
MYC
MYC
TUBA1A
D
Pan Kla
Hydroxy-HIF-1α
MYC
MYC
TUBA1A
G
Pan Kla
Hydroxy-HIF-1α
HIF-1α
VHL
HIF-1α
TUBA1A
J
MYC
Hydroxy-HIF-1α
VHL
MYC
TUBA1A
M
MYC
Pan Kla
VHL
MYC
TUBA1A
P
Pan Kla
Hydroxy-HIF-1α
MYC
MYC
TUBA1A

Fig. 6-2

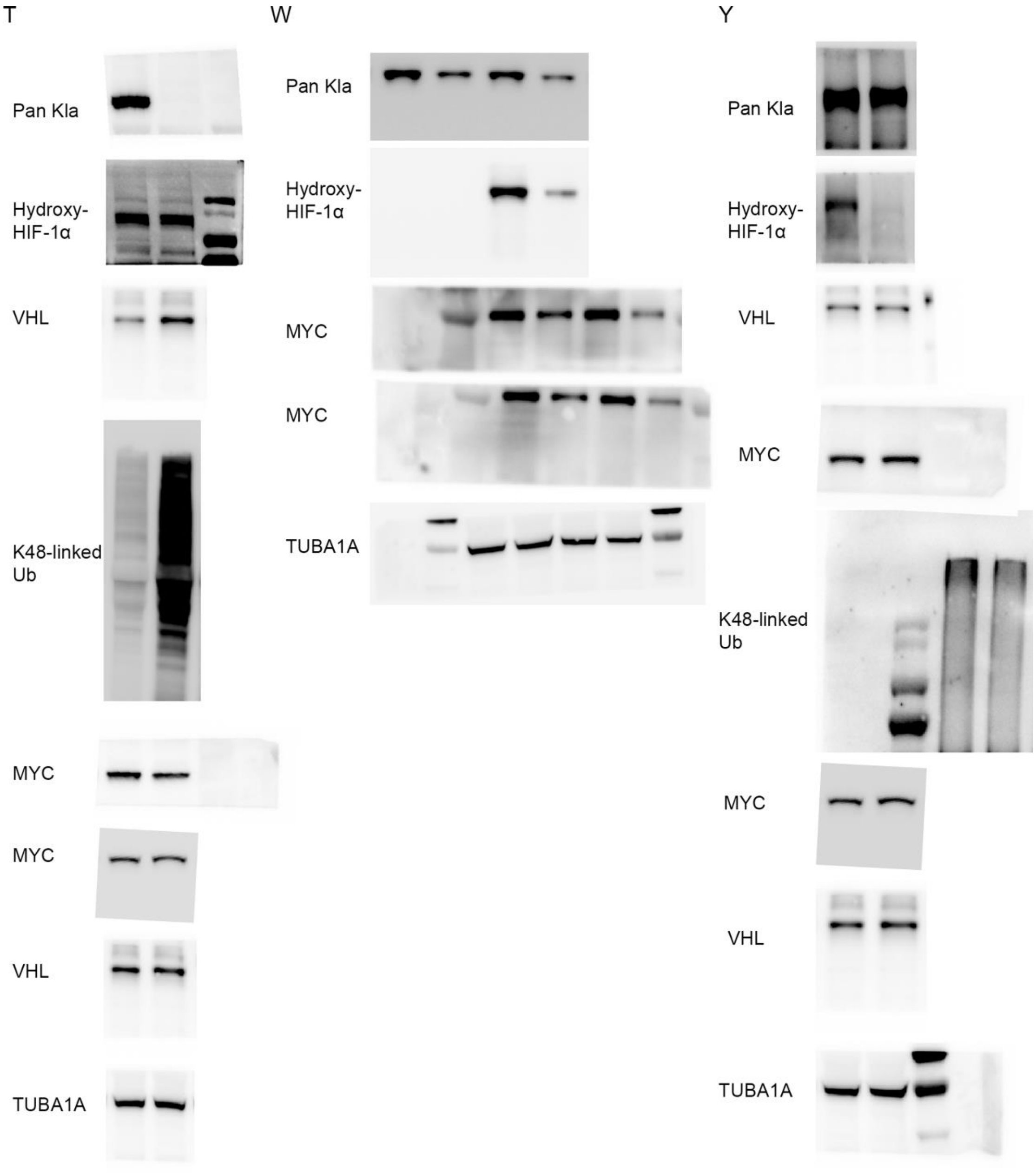
T
W
Y
Pan Kla
Hydroxy-HIF-1α
VHL
K48-linked Ub
MYC
MYC
VHL
TUBA1A
Pan Kla
Hydroxy-HIF-1α
MYC
MYC
TUBA1A
Pan Kla
Hydroxy-HIF-1α
VHL
MYC
K48-linked Ub
MYC
VHL
TUBA1A

Fig. 7

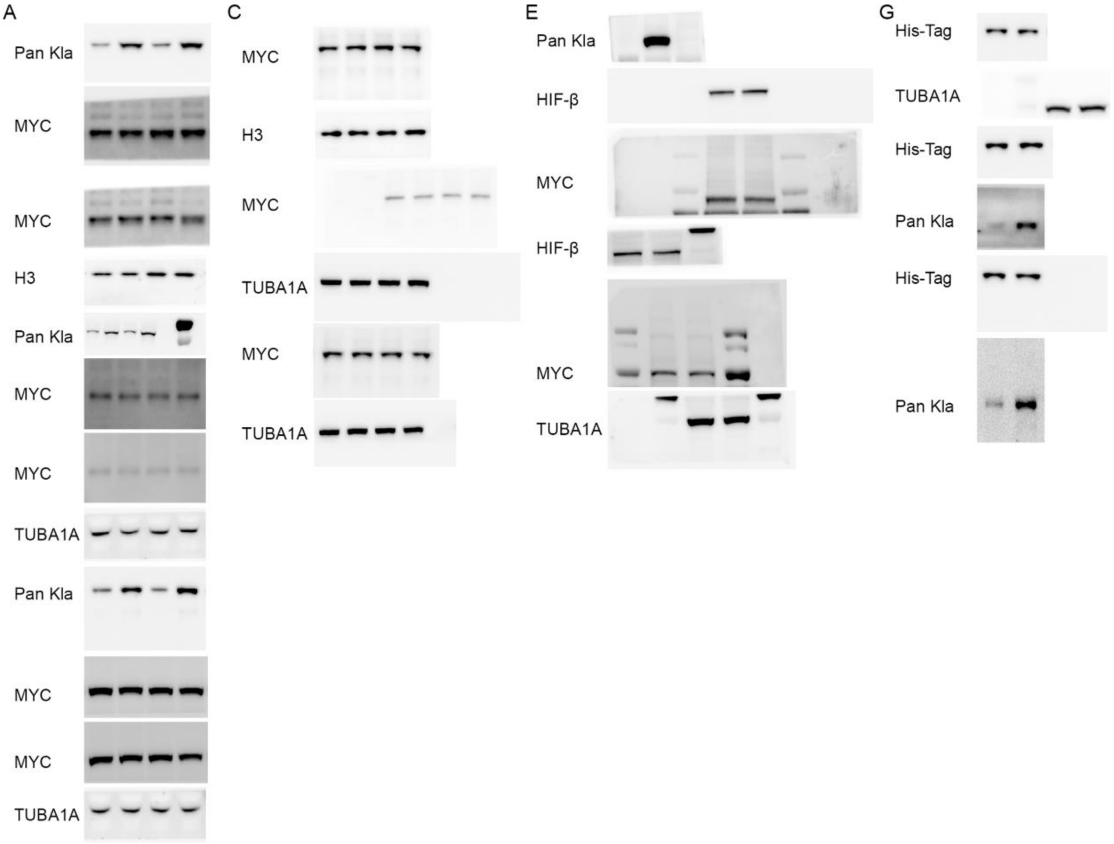
A
Pan Kla
MYC
MYC
H3
Pan Kla
MYC
MYC
TUBA1A
Pan Kla
MYC
MYC
TUBA1A
C
MYC
H3
MYC
TUBA1A
MYC
TUBA1A
E
Pan Kla
HIF-β
MYC
HIF-β
MYC
TUBA1A
G
His-Tag
TUBA1A
His-Tag
Pan Kla
His-Tag
Pan Kla

Fig. S1

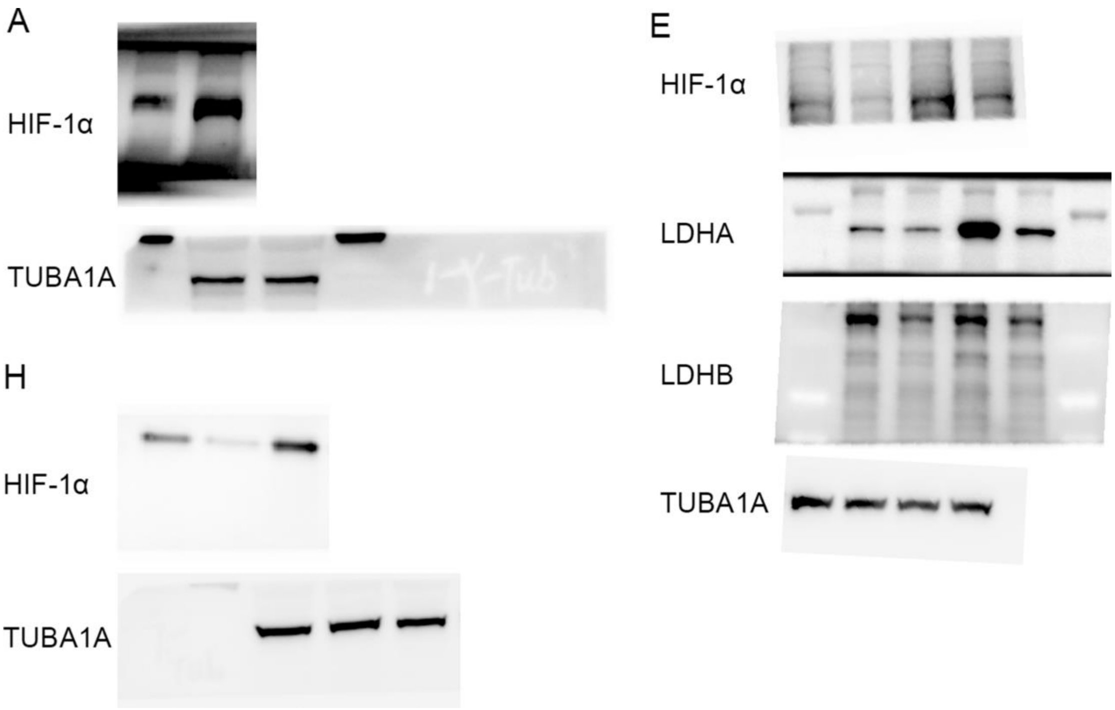
A
HIF-1α
TUBA1A
H
HIF-1α
TUBA1A
E
HIF-1α
LDHA
LDHB
TUBA1A

Fig. S2

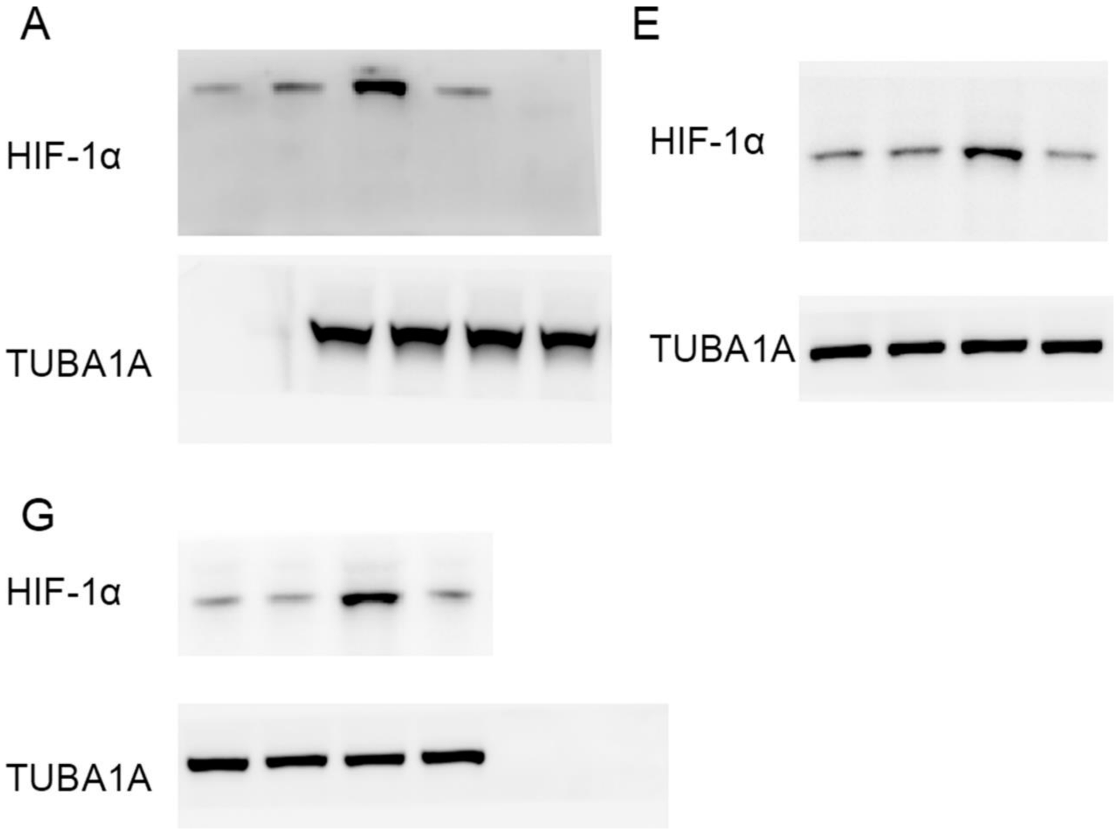

Fig. S3

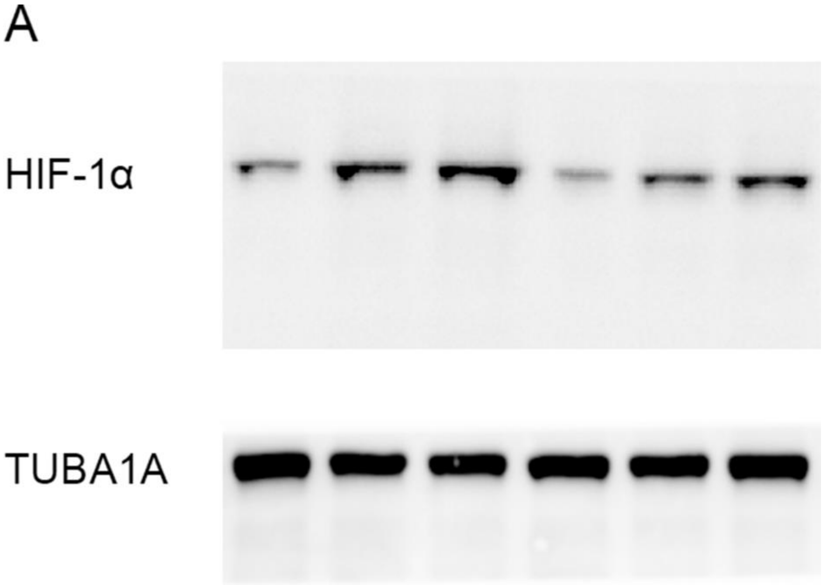

Fig. S4

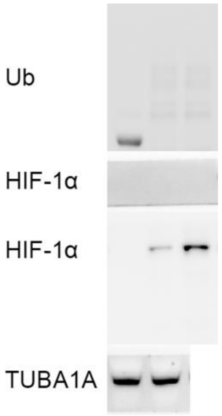
Ub
HIF-1α
HIF-1α
TUBA1A

Fig. S6

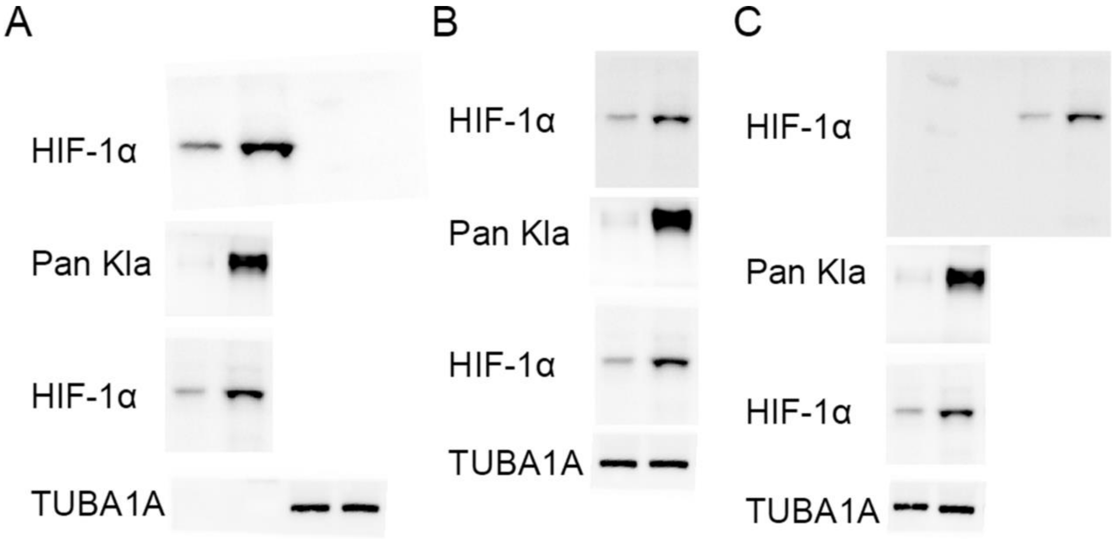
A
HIF-1α
Pan Kla
HIF-1α
TUBA1A
B
HIF-1α
Pan Kla
HIF-1α
TUBA1A
C
HIF-1α
Pan Kla
HIF-1α
TUBA1A

Fig. S7

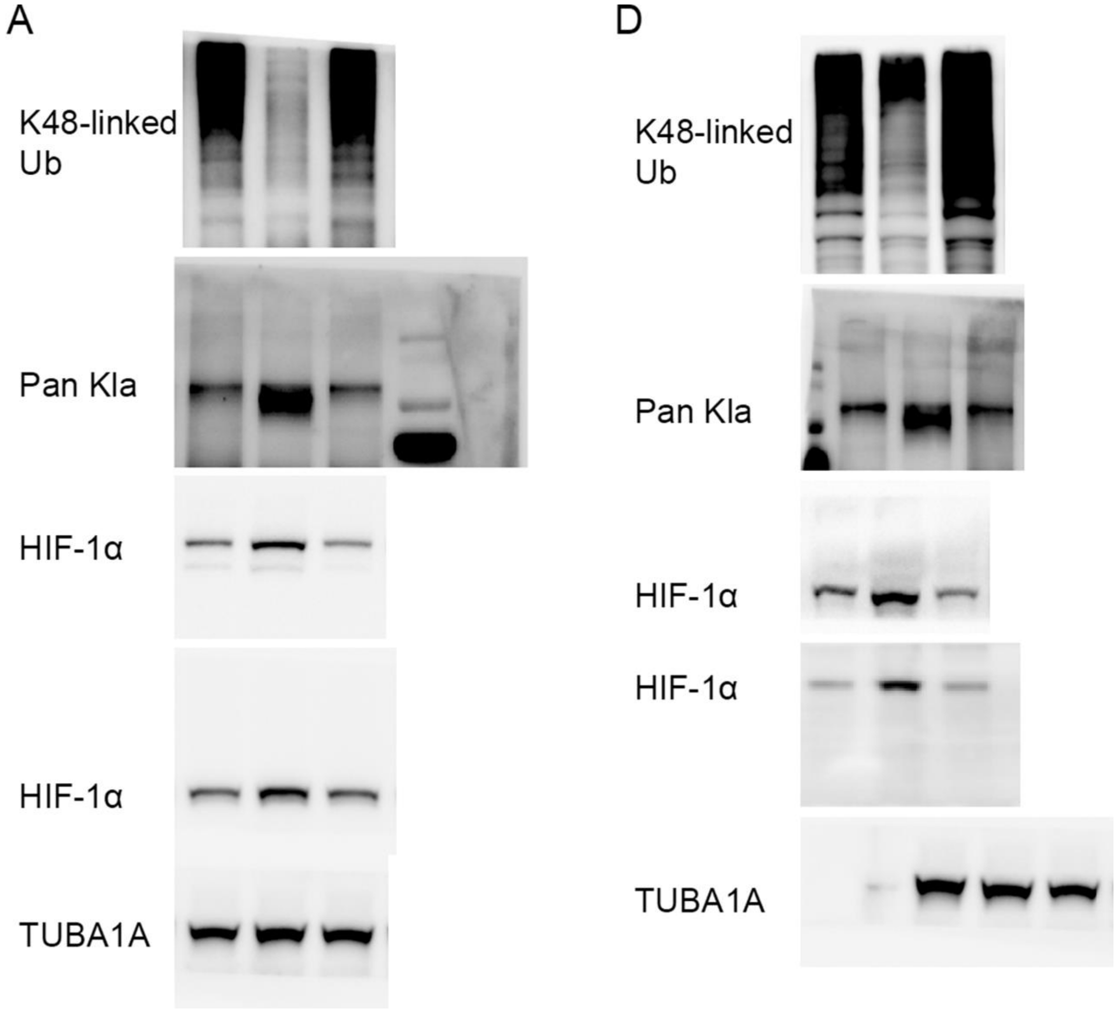
A
K48-linked Ub
Pan Kla
HIF-1α
HIF-1α
TUBA1A
D
K48-linked Ub
Pan Kla
HIF-1α
HIF-1α
TUBA1A

Fig. S8

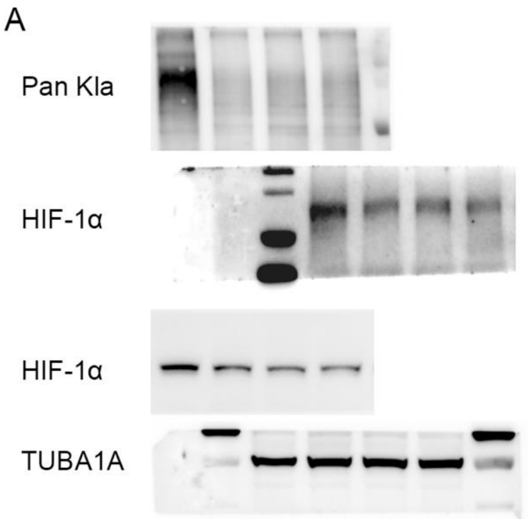
A
Pan Kla
HIF-1α
HIF-1α
TUBA1A

Fig. S10

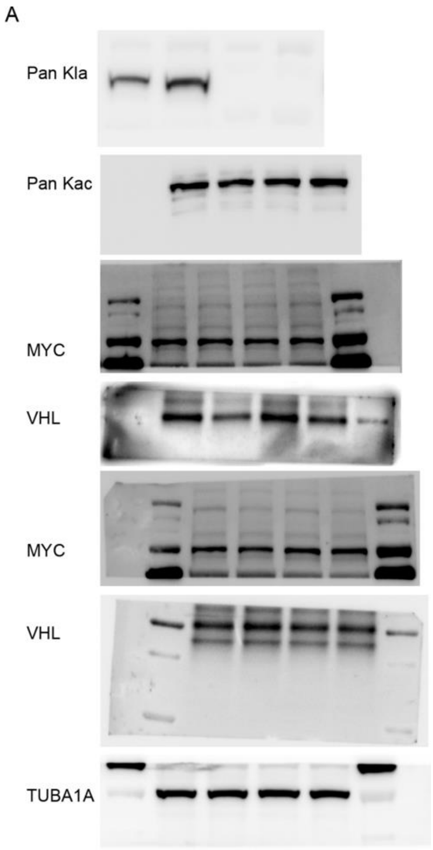

Fig. S11

B

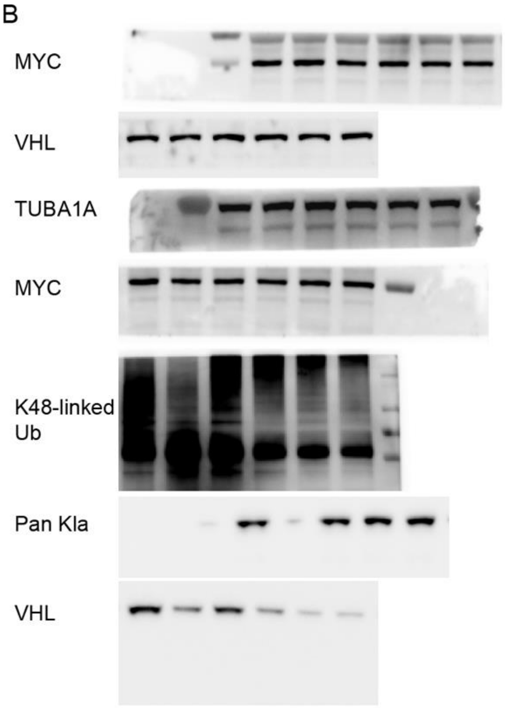
MYC
VHL
TUBA1A
MYC
K48-linked Ub
Pan Kla
VHL

H

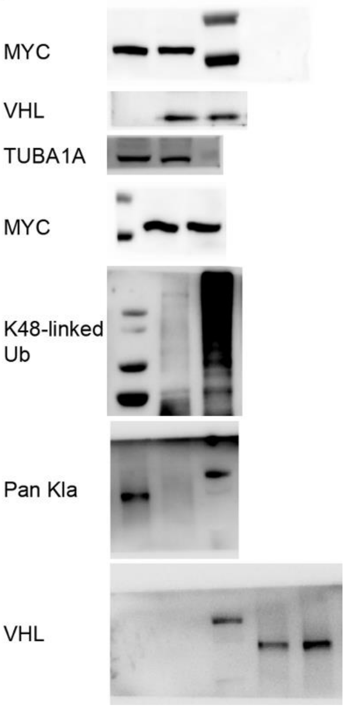
MYC
VHL
TUBA1A
MYC
K48-linked Ub
Pan Kla
VHL

Fig. S12

A

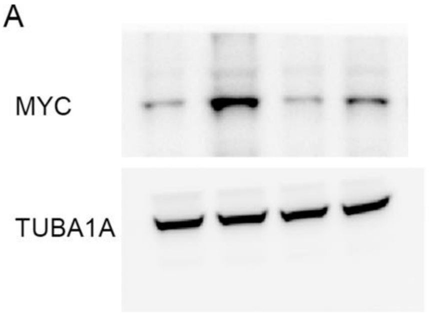
MYC
TUBA1A

C

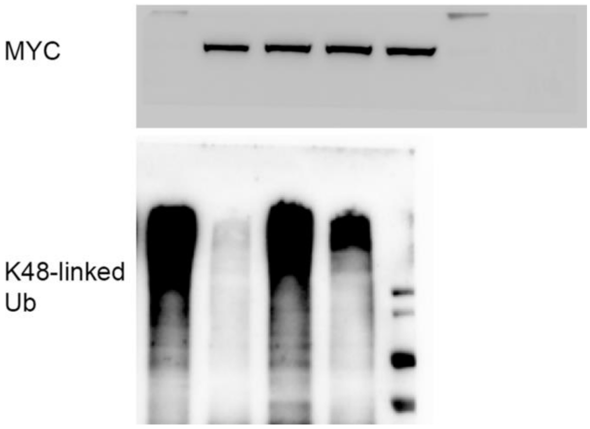
MYC
K48-linked Ub
